# A New Hybrid Neural Network Deep Learning Method for Protein–Ligand Binding Affinity Prediction and De Novo Drug Design

**DOI:** 10.3390/ijms232213912

**Published:** 2022-11-11

**Authors:** Sarita Limbu, Sivanesan Dakshanamurthy

**Affiliations:** Lombardi Comprehensive Cancer Center, Georgetown University Medical Center, Washington, DC 20057, USA

**Keywords:** protein–ligand binding affinity prediction, virtual screening, de novo drug design, machine learning, hybrid neural network, convolution neural network, fast forward neural network

## Abstract

Accurately predicting ligand binding affinity in a virtual screening campaign is still challenging. Here, we developed hybrid neural network (HNN) machine deep learning methods, HNN-denovo and HNN-affinity, by combining the 3D-CNN (convolutional neural network) and the FFNN (fast forward neural network) hybrid neural network framework. The HNN-denovo uses protein pocket structure and protein–ligand interactions as input features. The HNN-affinity uses protein sequences and ligand features as input features. The HNN method combines the CNN and FCNN machine architecture for the protein structure or protein sequence and ligand descriptors. To train the model, the HNN methods used thousands of known protein–ligand binding affinity data retrieved from the PDBBind database. We also developed the Random Forest (RF), Gradient Boosting (GB), Decision Tree with AdaBoost (DT), and a consensus model. We compared the HNN results with models developed based on the RF, GB, and DT methods. We also independently compared the HNN method results with the literature reported deep learning protein–ligand binding affinity predictions made by the DLSCORE, KDEEP, and DeepAtom. The predictive performance of the HNN methods (max Pearson’s R achieved was 0.86) was consistently better than or comparable to the DLSCORE, KDEEP, and DeepAtom deep learning learning methods for both balanced and unbalanced data sets. The HNN-affinity can be applied for the protein–ligand affinity prediction even in the absence of protein structure information, as it considers the protein sequence as standalone feature in addition to the ligand descriptors. The HNN-denovo method can be efficiently implemented to the structure-based de novo drug design campaign. The HNN-affinity method can be used in conjunction with the deep learning molecular docking protocols as a standalone. Further, it can be combined with the conventional molecular docking methods as a multistep approach to rapidly screen billions of diverse compounds. The HNN method are highly scalable in the cloud ML platform.

## 1. Introduction

Accurate methods of computing ligand affinity with a biological target are strongly desirable in drug discovery. Millions of compounds can be classified as ‘active’ and ‘inactive’ for a specific target using different virtual screening approaches. A ligand binds to the target protein and forms a protein–ligand complex that produces the effects such as inhibition and activation of a protein. Predicting the binding affinity and binding interaction strength between the ligand and the target protein is a key step in drug discovery. Determining the binding affinity helps identify the best ligand as the potential drug candidate. Computational approaches to predicting binding affinity instead of in vitro experiments significantly reduce drug discovery time and cost. The early phase of the small molecule drug discovery deals with the rational drug design for a protein target. Potential small molecules are selected as hits based on their binding affinity. Several computational methods have been developed to predict protein–ligand binding affinity [1,2,3,4,5,6,7]. However, these computational approaches make inaccurate modeling assumptions, resulting in poor predictability problems in the hit identification with high false-positive rates [1,2,3,4,5,6,7]. Further, in rank-ordering the hits in the virtual screening, reliance on docking scores that do not provide broadly reliable or valuable information that can be applied to hit prioritization and identification [1,2,3,4,5,6,7]. Therefore, accurately predicting the binding affinity and target hits using structure-based methods is still challenging.

The machine learning techniques use the experimentally derived protein–ligand data to learn and train their models to predict the binding affinity of a new protein–ligand complex; however, some challenges still remain, such as accurate treatment of protein–ligand interactions, protein flexibility, appropriate descriptors, and wide variations in the ligand affinity values [1,2,3,4,5,6,7,8,9]. Among the machine learning models, the random forest (RF) method improves affinity prediction compared to other existing methods [1,2,3,4,5,6,7,8,9] by developing models such as RF-Score and SFCscore^RF^. Deep learning models are popular due to their proven high performance in the field, such as visual data recognition [10], speech recognition [11], and drug discovery [12]. Several deep machine-learning methods have been proposed for ligand affinity prediction and de novo drug design. DEELIG [13] is a deep learning model developed by Ahmed et al. using convolutional neural networks (CNN) to find spatial relationships among data. It uses a 3D grid of atoms as features representing the protein–ligand complex. Li et al. [14] developed DeepAtom based on a CNN that extracts useful atom interaction features from the voxelized structure of a protein–ligand complex. K_DEEP_, developed by Jiménez et al. [15], uses the 3D voxel representation of both protein and ligand in their CNN-based model. Hassan et al. [16]. developed DLSCORE based on fully connected neural networks (FCNN) on a set of 348 BINANA descriptors, including protein–ligand interactions. DeepDTA [17] by Öztürk et al. used the protein sequence information, and SMILES representation of the ligands, to predict the drug target affinity using CNN. Many other deep-learning methods have been reported to predict protein–ligand binding affinity [18,19,20,21,22,23,24,25,26,27,28,29,30]. However, the explicit treatment and modeling of ligand properties using a separate learning framework that increases the prediction rate and accuracy are absent in the machine learning methods.

In this study, we developed a new hybrid neural network (HNN) deep learning model comprising the ‘HNN-denovo’ and ‘HNN-affinity’ methods that use a separate learning framework to increase prediction accuracy. The HNN model combines the CNN and the FCNN machine architectures to model the protein structure, protein–ligand interactions, protein sequences, and ligand descriptors. The HNN-denovo method uses the protein pocket structure and ligand interactions as input features, whereas the HNN-affinity method uses protein sequences as input features. In both cases, we modeled the ligands by the SMILES string, which is used for the ligand descriptor calculation. The ligand SMILES and descriptor features are modeled separately by the CNN framework. Overall, the HNN method consistently outperforms the ligand binding affinity prediction deep learning methods published in the literature.

## 2. Results and Discussion

**Figure 1:**
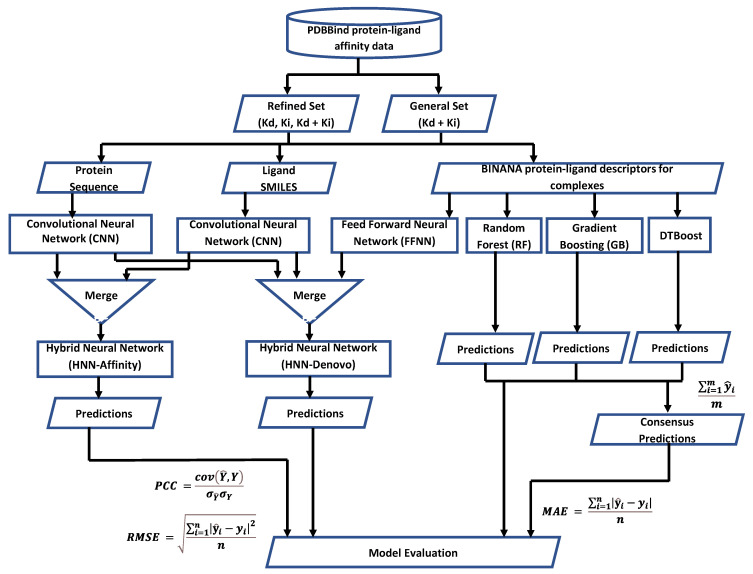
The overall workflow schema of experimental design, the dataset, training, and testing process of the deep learning-based hybrid neural network methods HNN-affinity and HNN-denovo and other machine learning models that we developed in this study.

**Figure 2:**
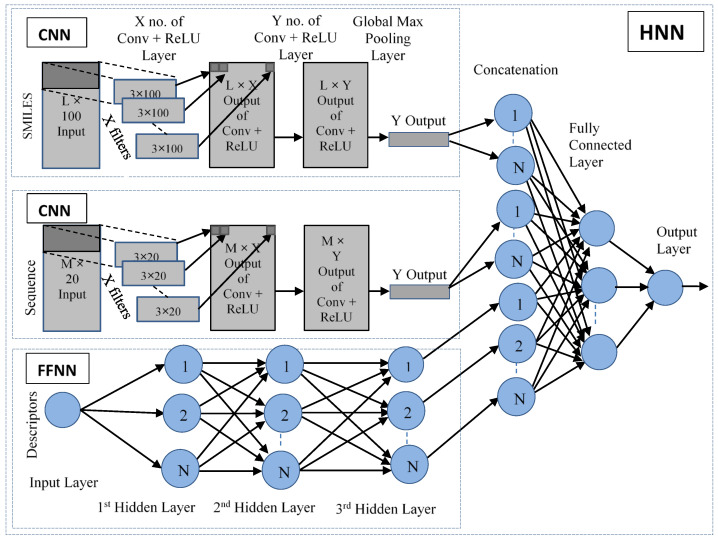
The deep learning-based hybrid neural network (HNN) model framework.

In this study, we developed two hybrid neural network deep learning methods, called ‘HNN-denovo’ and ‘HNN-affinity’ for the de novo design and to predict binding affinity. We applied the HNN methods to rapidly screen a precompiled known library of protein–ligand complexes and predicted their binding affinity. The overall schema workflow of the HNN method is displayed in Figure 1. The HNN method was developed as a hybrid deep neural network model in Python using the Keras API with Tensorflow in the backend (Figure 2). The HNN model consists of a convolutional neural network (CNN) for deep learning based on structure attributes (SMILES) and a multilayer perceptron (MLP)-type feed-forward neural network (FFNN) for learning based on the protein binding pocket, protein sequence, and protein–ligand interaction descriptors (Figure 1 and Figure 2). HNN-denovo uses both amino acid sequence and structure information of the protein and SMILES of the ligand for designing novel small molecules. The HNN-denovo method uses target protein binding pocket information such as ligand-binding site interactions, ligand-specific descriptors, and fingerprints. The protein and ligand structural interaction descriptors are calculated by BINANA [31], which examines the docked ligand poses inside the protein pocket to identify non-covalent and covalent molecular interactions such as hydrogen bonds, salt bridges, etc., and pi interactions that contribute to the ligand binding. The HNN-affinity method uses proteins and their binding pocket sequences for the CNN and ligand structure for the CNN. In both cases, SMILES of the ligands were used for the ligand descriptor calculations.

We used HNN learning than stacking learning for the following reasons. Stacking learning considers several models based on the different algorithms as the weak learner. Further, several models based on different algorithms are developed on the same training set in stacking, and the predictions are made for the same test set. Then the predictions are stacked and taken as the meta model’s input features to make a better final prediction. Whereas the HNN learning first makes models based on the CNN that considers ligand features and the FFNN that considers the protein feature descriptors. Then we concatenate the CNN and FFNN models but not their individual predictions to make the final prediction.

We included a new SMILES feature representation method in the HNN framework by modifying our previous 3D array representation of 1D SMILES simulated by the convolutional neural network (CNN) [32,33]. We assembled protein–ligand binding data such as K_d_, K_i_, and the combined K_d_ and K_i_ values from a general set and refined set obtained from the PDBBind database. To train the machine learning models, we computed a total of 653 protein- and ligand-specific molecular descriptors that were modeled by FFNN and the SMILES as ligand chemical features that are modeled by CNN. We also developed models based on RF, GB, and DT. We then predicted ligand binding affinity for the precompiled known library of protein–ligand complexes obtained from the PDBBind database. Since the HNN model suffers from higher variance in comparison to other machine learning methods, such as RF, we decided to make predictions multiple times and evaluated the performance based on the average of ten simulations. Though other machine learning methods, such as the RF method, has low variance with multiple simulations, to be consistent, we ran ten simulations for the RF, GB, and DT methods as well. The average of the RF, GB, and DT model predicted values were used for the consensus prediction. Next, we compared the HNN method-predicted protein–ligand affinity results with the RF, GB, and DT methods. We also compared the HNN-denovo and HNN-affinity performance metrics with the literature-reported deep learning methods such as DLScore, K-DEEP, and DeepAtom. The results are discussed in the following sections.

### 2.1. Model Evaluation

The performance of the models was evaluated as the average of 10 simulation results based on root mean squared error (*RMSE*), mean absolute error (*MAE*), and Pearson correlation coefficient (*PCC*). The MSE is the average of the squares of the errors, which are the differences between the predicted and actual values. The *MAE* is the average absolute difference between the predicted and the actual values. *PCC* measures the strength of the relationship between the two datasets of predicted and actual values.

$$RMSE=\sqrt{\frac{\sum_{i=1}^{n}{\left|{\hat{y}}_{i}-y_{i}\right|}^{2}}{n}}$$

$$MAE=\frac{\sum_{i=1}^{n}\left|{\hat{y}}_{i}-y_{i}\right|}{n}$$

$$PCC=\frac{cov\left(\hat{Y},Y\right)}{\sigma_{\hat{Y}}\sigma_{Y}}$$

where ${\hat{y}}_{i}$ is the predicted value of the *i*th dependent variable, $y_{i}$ is the *i*th observed dependent variable, $cov\left(\hat{Y},Y\right)$ is the covariance of the predicted and the observed data, and $\sigma_{\hat{Y}}\sigma_{Y}$ is the product of their standard deviation.

### 2.2. HNN-Denovo Method Validation

#### 2.2.1. Validation with PDBBind Protein–Ligand Complex Refined Set

**Models based on combined K_d_ and K_i_ binding data: Case 1 data set.**

**Figure 3:**
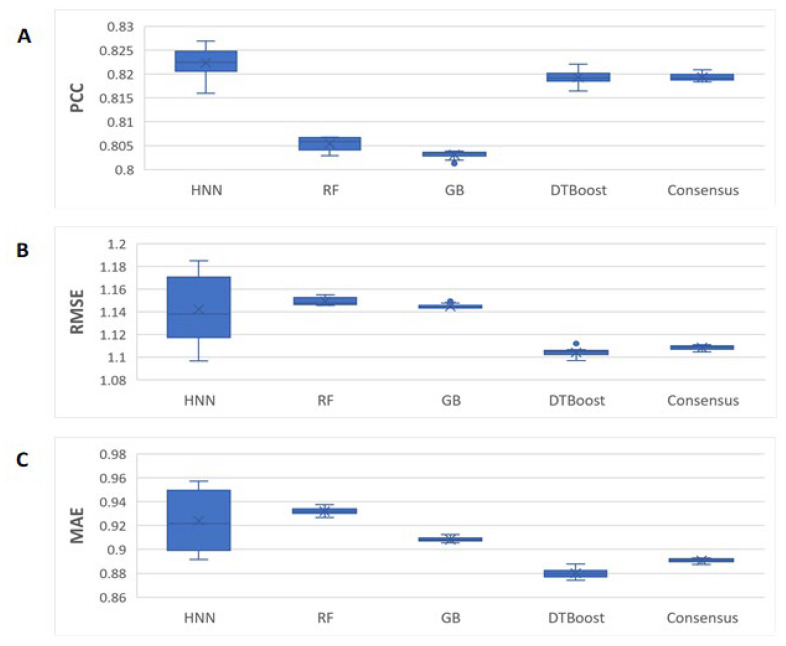
The HNN-denovo performance comparison to other models for the combined K_d_ and K_i_ binding data. (**A**) PCC, (**B**) RMSE, and (**C**) MAE of the binding affinity prediction by HNN, RF, GB, DT, and consensus methods.

The case 1 binding data (see methods section) is the combination of the K_d_ and K_i_ binding affinity data compiled with 4850 protein–ligand complexes. The models were developed with 3925 complexes in a training set and 925 complexes in the test set. The HNN-denovo model input features are the descriptors that represent the protein binding pocket structure and protein–ligand interactions computed by the BINANA algorithms [31], and the SMILES of the ligand. We implemented only BINANA descriptors as the input features for the RF, GB, and DTBoost, as they cannot handle SMILES input features. The PDB code of the complexes used in the training and test sets is provided in the Supplementary Material. The statistical performance metrics reported were averages of the 10 neural network simulations. The Pearson correlation coefficients (PCCs) for the HNN, RF, GB, DT, and consensus methods were 0.82, 0.80, 0.80, 0.81, and 0.81, respectively (Figure 2). The RMSEs were 1.142, 1.149, 1.144, 1.104, and 1.108, and the MAEs were 0.924, 0.932, 0.908, 0.879, and 0.89 for the HNN, RF, GB, DT, and consensus methods, respectively. The HNN-denovo model consistently performed equally or better than the RF, GB, and DT methods (Figure 3). The root mean square errors (RMSEs), i.e., binding affinity value prediction errors, and mean absolute errors (MAEs), i.e., the average magnitudes of the errors in the binding affinity value predictions, among the models were similar. Overall, for the combined K_d_ and K_i_ binding affinity data, the HNN-denovo consistently performed equally or better than the other machine learning methods (Figure 3).

**Models based on K_d_ binding data: Case 2 data set.**

**Figure 4:**
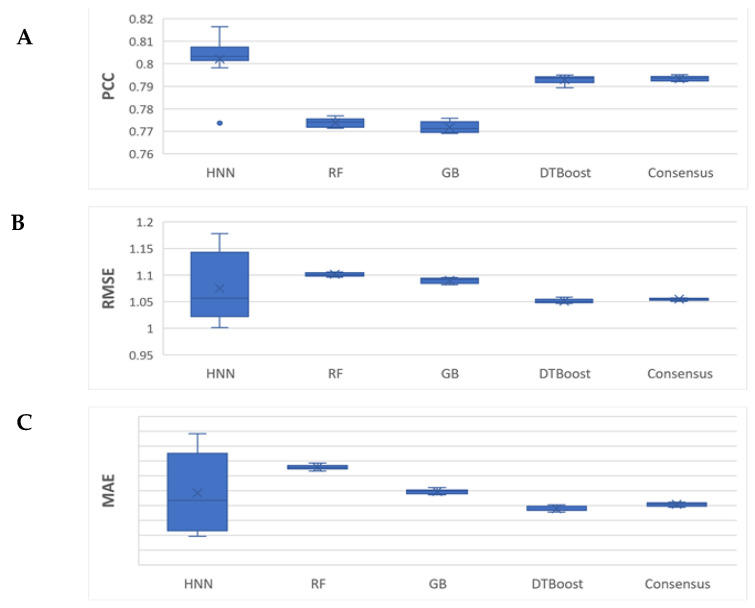
The HNN-denovo performance comparison to other models for the combined K_d_ binding data. (**A**) PCC, (**B**) RMSE, and (**C**) MAE of the binding affinity prediction by HNN, RF, GB, DT, and consensus methods.

Next, we sought to test the binding affinity prediction for a smaller data set. We considered K_d_ and K_i_ binding inhibition data in this case as separate data sets, case 2 and case 3, respectively. The case 2 binding data set (see methods section) comprised of K_d_ binding affinity data compiled with 2464 protein–ligand complexes. The models were developed on the 2004 complexes with K_d_ values in the training set, and the test set contained 460 complexes for which binding affinity was predicted. The HNN-denovo model used 348 BINANA protein–ligand interactions descriptors, protein pocket structure, and the SMILES of the ligands as input features, whereas RF, GB, and GT models used BINANA descriptors as the input features. The PDB codes of the complexes in the training and test sets for case 2 are provided in the Supplementary File. The statistical performance metrics reported were the averages of the 10 neural network simulations. The HNN, RF, GB, DT, and consensus methods predicted the binding affinity with an average PCC of 0.80, 0.77, 0.77, 0.79, and 0.79 (Figure 4). Notably, the protein–ligand binding affinity prediction correlation of the HNN-denovo for K_d_ binding data is optimal or higher than the RF, GB, and DT (Figure 4). The RMSE of the predictions was 1.075, 1.101, 1.09, 1.051, and 1.054, and the MAE was 0.857, 0.891, 0.859, 0.836, and 0.841 for the HNN, RF, GB, DT, and consensus methods, respectively. Taken together, for K_d_ data, the HNN-denovo model outperformed the other machine learning methods RF, GB, and DT (Figure 4). Evidently, the Root Mean Square Error (RMSE) and Mean Absolute Error (MAE) of the binding affinity predictions among the models are similar.

**Models based on K_i_ binding data: Case 3 data set.**

**Figure 5:**
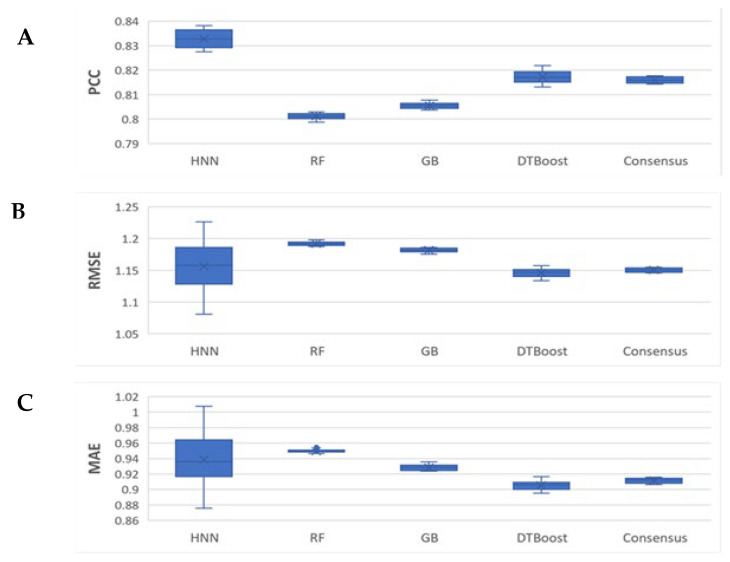
The HNN-denovo performance comparison to other models for the K_i_ binding data. (**A**) PCC, (**B**) RMSE, and (**C**) MAE of the binding affinity prediction by HNN, RF, GB, DT, and consensus methods.

Next, we sought to test the binding affinity prediction with K_i_ binding data. The case 3 binding data set (see methods section) comprised the K_i_ binding affinity data compiled with 2386 protein–ligand complexes. The models were developed for the 1936 complexes with K_i_ values in the training set, and the binding affinity was predicted for the 450 complexes in the test set. The HNN-denovo model used 348 BINANA descriptors, protein pocket structure, and the SMILES of the ligands as input features, whereas RF, GB, and DT models used only BINANA descriptors as the input features. The PDB codes of the complexes in the training and test sets for case 3 are provided in the Supplementary File. The statistical performance metrics reported were the averages of the 10 neural network simulations. The predicted ligand affinity correlation metric PCCs were 0.83, 0.80, 0.80, 0.82, and 0.81 for the HNN, RF, GB, DT, and consensus methods, respectively (Figure 5). The RMSEs were 1.156, 1.192, 1.182, 1.146, and 1.15, and the MAEs were 0.938, 0.95, 0.928, 0.905, and 0.911 for the HNN, RF, GB, DT, and consensus methods, respectively. Again, for the K_i_ data, the HNN-denovo model performed optimally in predicting the ligand binding affinity, while the RMSE and MAE, among the models, are similar (Figure 5). The root mean square errors (RMSEs) and mean absolute errors (MAEs) in the binding affinity value predictions, among the models, were similar.

#### 2.2.2. Validation with PDBBind Protein–Ligand Complex General Set

**Figure 6:**
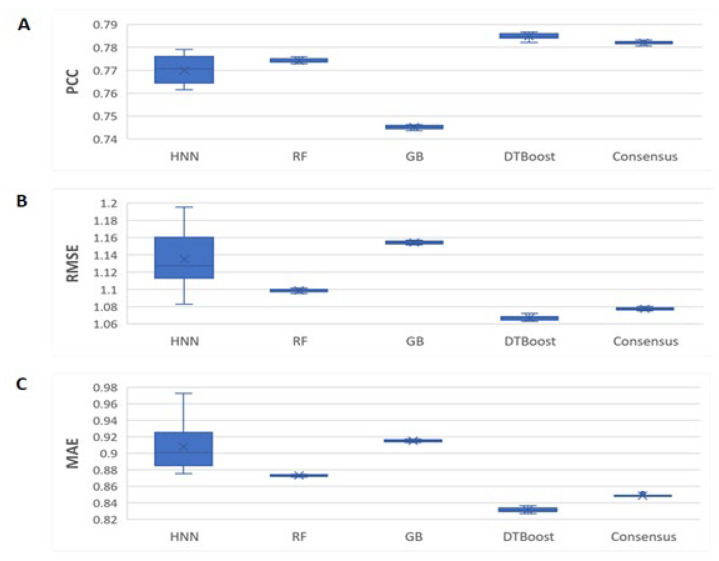
The HNN-denovo performance comparison for the general set combined ligand K_d_ and K_i_ binding data. (**A**) PCC, (**B**) RMSE, and (**C**) MAE of the binding affinity prediction by the HNN-denovo, RF, GB, DT, and consensus methods.

The next validation set we used is the PDBbind v2019 general set consisting of binding affinity data for 12,800 protein–ligand complexes. We compiled and combined K_d_ and K_i_ data in this general set and obtained a total of 6081 complexes. We processed and computed 358 protein–ligand complexes and ligand SMILES descriptors. The models were developed with the 5031 complexes in the training set, and the binding affinity was predicted for the 1050 test set complexes. Here, the HNN-denovo model used 348 BINANA protein–ligand interactions descriptors, protein pocket structure, and the SMILES of the ligands as input features, whereas RF, GB, and DT models used only the BINANA descriptors as the input features. The predicted ligand affinity correlation metric PCCs were 0.77, 0.77, 0.74, 0.78, and 0.78 for the HNN, RF, GB, DT, and consensus methods, respectively (Figure 6). The RMSEs were 1.134, 1.098, 1.154, 1.066, and 1.077, and the MAEs were 0.908, 0.873, 0.915, 0.831, and 0.848 for the HNN, RF, GB, DT, and consensus methods, respectively. The HNN-denovo model performed equally compared to the RF, and GB, with DTBoost performing slightly higher prediction accuracy (Figure 6). The root mean square errors (RMSEs) and mean absolute errors (MAEs) among the models were similar.

#### 2.2.3. Validation with PDBBind Protein–Ligand Complex Refined General Set

**Figure 7:**
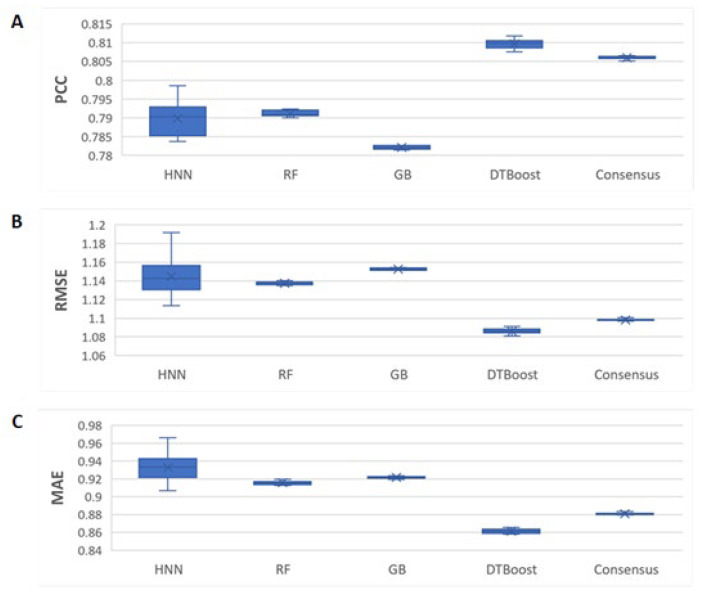
The HNN-denovo performance comparison to other models for the refined general set binding data. (**A**) PCC, (**B**) RMSE, and (**C**) MAE of the binding affinity prediction by the HNN, RF, GB, DT, and consensus methods.

We next sought to test the PDBBind refined general data set. It contains 10,931 complexes, with 4085 complexes obtained from the refined set and 6081 obtained from the general set. We used 8931 complexes in the training set and randomly selected 2000 complexes were used as the test set. The binding affinity was predicted for these 2000 complexes in the test set. The HNN-denovo model used 348 BINANA descriptors, protein pocket structure, and the SMILES of the ligands as input features, whereas RF, GB, and DT models used only the BINANA descriptors as the input features. The PDB codes of the complexes used in the training and test sets are provided in the Supplementary Material. The statistical performance metrics were reported as the average of the ten neural network simulations. The computed PCCs were 0.79, 0.79, 0.78, 0.81, and 0.81 for the HNN, RF, GB, DT, and consensus methods, respectively (Figure 7). The RMSEs were 1.144, 1.137, 1.152, 1.086, and 1.098, and the MAEs were 0.932, 0.915, 0.921, 0.861, and 0.88 for the HNN, RF, GB, DT, and consensus methods, respectively. The HNN-denovo model had optimal performance among the other machine learning methods, except DTBoost, which performed slightly better in this data set (Figure 7). The root mean square errors (RMSEs), i.e., binding affinity value prediction errors, and mean absolute errors (MAEs), i.e., the average magnitudes of the errors in the binding affinity value predictions, among the models were similar.

### 2.3. HNN-Affinity Predictions Based on the Protein Sequence and the Ligand SMILES as Input Features

Next, we developed the HNN-affinity to use only protein binding pocket amino acid sequences instead of protein structural features and Ligand SMILES as input features. The protein sequence-based ligand affinity prediction is useful when the protein structural complex is unavailable. The binding pocket amino acid sequences that are number encoded (see methods section) were used as input for the CNN, and Ligand SMILES were used as input for the FFNN. We used the BINANA descriptors as the input features for the RF, GB, and DTBoost models, as they cannot handle SMILES input features. We used the case 1 binding data of the PDBbind v2019 refined set (see methods section), which is the combination of the K_d_ and K_i_ binding affinity data compiled with a total of 4850 protein–ligand complexes. The models were developed with 3860 complexes in the training set, and the ligand binding affinity was predicted for the 990 complexes in the test set. The PDB codes of the complexes used in the training and test sets are provided in the Supplementary Material. The statistical performance metrics reported were the averages of the 10 neural network simulations. We compared the HNN-affinity results with HNN-denovo and the RF, GB, and DTBoost models (Table 1). The HNN-affinity and HNN-denovo predictive performances are close to each other. The HNN-denovo predicted with ~2% higher accuracy with the inclusion of BINANA descriptors. The binding affinity value prediction errors (RMSE) and the average magnitude of the errors (MAE) in the binding affinity value predictions among the models were similar. Evidently, the HNN-denovo performed well compared to the HNN-affinity, RF, GB, and DTBoost. The inclusion of protein and ligand structural, and protein–ligand interaction features contributed to the increased prediction accuracy of the HNN-denovo.

### 2.4. Comparative Performance of HNN-Denovo and HNN-Affinity to the Literature Reported Deep Learning Methods

Next, we sought to evaluate the performance of our HNN methods, with respect to the literature-reported ligand affinity prediction deep learning methods [13,14,15,16]. We compared the HNN-affinity and HNN-denovo results with the protein–ligand binding affinity prediction deep machine learning methods reported in the literature, such as DeepAtom [13,14], KDEEP [15], and DLScore [16]. The results of the HNN-denovo and HNN-Affinity performance metrics PCC and RMSE, with respect to the other deep learning methods DeepAtom [13,14], KDEEP [15], and DLScore [16], for the PDBbind 2019 refined set are presented in Table 2. Hassan et al. [16] reported a PCC 0.82 for the DLSCORE method, 0.21 for NNScore 2.0, and 0.15 for AutoDock Vina for the PDBbind v2016 data with 3199 complexes in the training set and 300 complexes in the test set. The RMSE reported was 1.15, 2.78, and 3.17, and the MAE was 0.86, 2.03, and 2.5 for the DLSCORE, NNScore 2.0, and Vina, respectively. Jiménez et al. [15] reported the PCC 0.82 with RMSE 1.27 for the KDEEP method, for the PDBbind v2016 data with 3767 complexes in the training set and 290 complexes in the test set. Li et al. [14] reported that the PCC 0.81 with RMSE was 1.31 for the DeepAtom method. Li et al. used the training set containing 3390 complexes, and 377 complexes in the test set, obtained from the PDBbind v2016 data. Further, the same author, Li et al. [14], reported the PCC 0.83 with RMSE 1.23 for the DeepAtom method, with a large training set consisting of 9363 complexes and the test set with 1000 complexes, a combined data obtained from the PDBbind v2016 plus MOAD database. In contrast, with only 3860 complexes in the training set, the HNN-affinity achieved a higher PCC of 0.83 and 0.82 and the RMSE 1.04 and 1.06 for the unbalanced (i.e., a smaller number in the test data set) data of 300 complexes and balanced test data set of 797 complexes, respectively (Table 2). Similarly, for the 4357 complexes in the training set, with the same test set size of 300 and 797 unbalanced and balanced test data sets, our HNN-denovo achieved a higher PCC of 0.86 with RMSE 1.11 and PCC 0.84 with RMSE 0.98, respectively (Table 2). Notably, even with the higher training to test set ratios (15.4 and 17.1), our HNN-denovo method outperformed other deep learning methods in the binding affinity prediction rate (PCC 0.82 and 0.84), i.e., correctly predicting the native ligands for a given test protein (Table 2). The unbalanced data set usually boosts the prediction performance more than the balanced data set, which is reflected in our method predictions. This is also true for the other deep learning methods, such as the KDEEP and DeepAtom methods that used a small number of data sets, ~10%, in their test set (Table 2). Further, the HNN-denovo achieved with the highest prediction rate with a PCC of 0.86, whereas it was 0.83 for the HNN-affinity. Taken together, our HNN methods consistently performed better than or equally well compared to other deep learning methods for the balanced and unbalanced data sets.

## 3. Methods and Materials

### 3.1. Data Sets

The ‘refined set’ and the ‘general set’ consisting of experimentally measured binding affinity data for biomolecular protein–ligand complexes reported in the Protein Data Bank (PDB) were downloaded from the PDBbind database [34].

Refined Set: The refined set from PDBbind v2019 consists of 4852 protein–ligand complexes annotated with the ligand affinity data. The ligand affinity data were provided as K_d_ or K_i_ inhibition binding constant values. From a total of 4850 protein–ligand complexes, 2464 complexes with K_d_ data and 2386 complexes with K_i_ data were processed to calculate the protein and ligand-centric descriptors of the complexes. The binding affinity K_d_ and K_i_ are converted to pK_d_ and pK_i_ (logarithm of the inverse of K_d_ and K_i_) to predict-ligand affinity (pK_d_ or pK_i_) using machine learning methods.

Case 1. The case 1 data set consists of a combined K_d_ and K_i_ binding affinity data with a total of 4850 protein–ligand complexes. Models were developed with a training set 3925 protein–ligand complexes containing both K_d_, and K_i_ data, and 925 complexes were used as a Test set.
Case 2. The case 2 data set consists of 2464 protein–ligand complexes with K_d_ data. Models were developed with a training set of 2004 protein–ligand complexes with K_d_ binding affinity data, and the remaining 460 complexes were used as a test set.
Case 3. The case 3 data set consists of 2386 protein–ligand complexes with K_i_ data. Models were developed with a training set of 1936 protein–ligand complexes with K_i_ binding affinity data, and 450 complexes were used as a test set.

General Set: The PDBbind v2019 general set consists of binding affinity data for 12,800 protein–ligand complexes. The K_d_ and K_i_ data were processed and combined and the K_d_ and K_i_ data and obtained a total of 6081 protein–ligand complexes. Models were developed with 5031 complexes in the training set and 1050 complexes in the test set.

Refined-General Set: Refined-General Set contains a total of 10,931 complexes. The refined-general set was obtained by combining 4085 complexes from the refined set and 6081 complexes from the general set. We used 8931 complexes for the training set, and 2000 complexes were randomly selected for the test set.

### 3.2. Calculation of Protein and Ligand Features

#### 3.2.1. Computation of BINANA Descriptors as Protein-Ligand Interaction Structural Features

We used the protein pocket structure, and protein-ligand interactions modeled by BINANA descriptors as input features for the HNN-denovo method. Additionally, for the HNN-denovo method, protein sequences (described in the section below) were used as additional features. The protein and ligand files in .pdb and .mol2 format were first converted to .pdbqt format by prepare_receptor4.py and prepare_ligand4.py, respectively, MGLTools [35]. The BINANA (BINding ANAlyzer) algorithm [31] was used to calculate the descriptors of the protein-ligand complexes for the proteins and ligands in .pdbqt format. A total of 348 BINANA descriptors for each protein and its ligand were calculated, including covalent and noncovalent interactions such as electrostatic interactions, ligand atom types, rotatable bonds, salt bridges, hydrogen bonds, ‘π’ interactions, and binding-pocket flexibility.

#### 3.2.2. SMILES as Ligand Features

For the deep learning method, SMILES were used as one of the features of the protein-ligand complexes. The SMILES of the ligands were calculated from their .mol2 files using Schrodinger’s structconvert utility. SMILES are in text format and were encoded as numbers to be used as input for the CNN. Unique indexes were created for 94 characters from ‘!’ to ‘~’ in the ASCII table to represent all possible SMILES characters in any format. The mapping of each character to a unique index was stored in python’s dictionary Dsmi = {‘!’:1, ‘”’:2, ‘#’:3, ‘$’:4, …, ‘C’:35, …, ‘~’:94} and made the vocabulary of possible characters in the SMILES. The SMILES of each ligand were encoded with unique numbers and either truncated or padded with 0s to the maximum allowed length L. The SMILES for the input compounds were converted to a 2D matrix of size K × L, where K was the number of input SMILES, and L = 325 was the allowed maximum length of the SMILES string used in the model.

#### 3.2.3. Protein Pocket Sequence as Input Features

We also used protein pocket sequences as input features for the HNN-affinity method. The sequences of the amino acids were extracted from the files in the .pdb format provided by PDBbind for the binding pockets of the protein-ligand complexes. The pocket sequences, also in text format, were number-encoded to be used as input for the CNN. Each of the 20 alphabetical characters representing the 20 amino acids was mapped to a unique index by a dictionary, Dseq = {‘A’:1, ‘R’:2, …, ‘V’:20}. The sequence of each pocket was encoded with unique numbers and either truncated or padded with 0s to the maximum allowed length M. The sequences were converted to a 2D matrix of size K × M, where K was the number of input sequences, and M = 150 was the allowed maximum length of the sequence used in the model.

### 3.3. Machine Learning Models

#### 3.3.1. Hybrid Neural Network Model (HNN)

The deep learning-based hybrid neural network model (Figure 2) was developed using Keras API in python with Tensorflow in the backend. The HNN model consists of two convolutional neural networks (CNNs) and one multilayer perceptron (MLP) type fully connected neural network (FCNN). One CNN (CNN-1) was used for training the model based on the structure of the ligand in each complex using the number-encoded Ligand SMILES of length L = 325. The second CNN (CNN-2) was used for training the model based on the protein pocket structural sequence in each complex using the number-encoded pocket sequence of length M = 150. The MLP-type FCNN was used for training the model based on 348 BINANA descriptors that characterized the protein-ligand interactions. The L length number-encoded SMILES was converted to a dense vector of size L × 100. The M length number-encoded pocket sequence was converted to a dense vector of size M × 20 by the embedding layer of Keras. The CNNs used Conv1D convolution with a filter of size 3 to extract features from the inputs. The model used the ReLU activation function and L2 regularization for both types of networks. Dropout and L2 regularization were used to prevent the model from overfitting. GlobalMaxPooling1D was used in the CNNs to downsample the feature maps. The output of the pooling layer of CNNs and the output of the FCNN were merged and processed by the final fully connected layer to predict the outcome using the linear activation function.

#### 3.3.2. Parameter Optimization

Hyperparameter optimization was implemented using the tree-structured Parzen estimator (TPE) method within the hyperopt package of python to search for optimum parameters to improve the model’s performance (Table 3). This approach requires defining the objective function that the fmin() function minimizes, the parameter space over which the search is performed, and the maximum number of evaluations to run.

#### 3.3.3. Other Machine Learning Algorithms

To compare the HNN-affinity prediction results, we also developed regression models based on machine learning methods based on the random forest (RF), gradient boosting (GB), and decision tree with AdaBoost (DT). A consensus prediction was made based on the average prediction rate of the three methods, excluding the HNN.

## 4. Conclusions

In this study, we developed, implemented, and validated the HNN-denovo and HNN-affinity for the ligand binding affinity prediction in the de novo design campaign. The HNN-denovo and HNN-affinity confer a higher prediction rate and most of the time successfully retrieve the cognate ligands for the proteins. The present versions of the HNN-denovo method reached the highest prediction accuracy, with a Pearson correlation coefficient (PCC) of 0.86, i.e., 86% of the time, it correctly predicts the native ligands based on the ~9000 protein-ligand complexes obtained from the PDB-Bind database. The HNN-denovo can generate small molecule hits specific to a protein binding site under the guidance of a drug-target binding affinity prediction structure model. The HNN-affinity can generate small molecule hits specific to a protein binding site under the guidance of protein binding site amino acid sequence patterns. The known limitation of the structure-based machine learning methods in predicting ligand binding affinity is that they require a protein-ligand experimental structural complex or an accurate docked ligand structural complex. The absence of such a protein-ligand structural complex or inaccurate structural complex will lead to an inaccurate ligand affinity prediction. Consequently, a machine learning prediction method that includes the protein sequence-based prediction framework is needed. Our HNN-affinity method can predict ligand affinity based on the protein amino acid sequence as input features in the absence of the protein structural information. Notably, HNN-affinity is particularly useful when the protein-ligand complex structure is unavailable; when dealing with undruggable targets; and when targeting intrinsically disordered proteins. Further, the HNN-affinity can predict different binding affinities such as *K_D_*, *K_I_*, and IC_50_ as it is trained on thousands of known protein-ligand complexes with known *K_D_*, *K_I_*, and IC_50_ values.

In summary, we have demonstrated that the HNN method shows promising performance for diverse data sets and outperforms the other deep learning methods for balanced and unbalanced data sets. The promising results on various datasets demonstrated that our HNN method could be potentially implemented in combination with molecular docking-based virtual screening campaigns, particularly when screening billions of compounds. We are adapting the HNN method with the molecular docking-based large-scale virtual screening pipeline. The HNN method is highly scalable and uses Keras and Tensorflow to generate and train the neural networks. The HNN virtual screening simulations can be performed with high-performance GPUs on the cloud to screen billions of diverse compounds rapidly. We ran it in our powerful GPU Linux server, which is highly scalable to run on the cloud ML platform to reduce computational time. Thus, our HNN method is scalable with high-performance GPU computing on Cloud to screen billions of diverse compounds rapidly.

## 5. Limitations

The HNN is complex when applied with a very large number of parameters for CNN and FFNN. As a result, the HNN method suffers from higher variance in comparison to other machine learning methods such as RF, which are visible in the box plots in Figure 3, Figure 4, Figure 5, Figure 6 and Figure 7 as they have a wider interquartile range (IQR). Further, in this study, we did not apply the statistical test of Chi-square conformity, because the binding affinity predicting models give continuous outcomes. In contrast, Chi-square tests are applied for data with the categorical outcome.

## Figures and Tables

**Table 1:** The comparative results of the HNN-denovo, and HNN-affinity.

	HNN-Affinity	HNN-Denovo	RF	GB	DTBoost	Consensus
	Protein Sequences + SMILES	Protein Sequences + SMILES + BINANA Descriptors	BINANA Descriptors Only			
RMSE	1.04	0.98	0.99	1.00	0.95	0.95
MAE	0.87	0.79	0.81	0.80	0.76	0.77
PCC	0.82	0.84	0.82	0.81	0.83	0.83
MSE	1.09	0.97	0.98	1.01	0.91	0.91

**Table 2:** The predictive performance (PCC and RMSE) comparison of the HNN-affinity, HNN-denovo, and the literature reported deep learning methods for the PDBbind 2019 refined set.

Machine Learning Method	Training/Test Data Protein–Ligand Complex	PCC	RMSE	Training to Test Set Ratio (%) from the Total # Complexes
HNN_affinity_ (Sequence+SMILES)	3860/300 (PDBbind 2019)	0.83	1.04	7.2
HNN_denovo_ (Sequence+Structure+SMILES)	4357/300 (PDBbind 2019)	0.86	1.11	6.4
HNN_affinity_ (Sequence+SMILES)	3860/797 (PDBbind 2019)	0.82	1.06	17.1
HNN_denovo_ (Sequence+Structure+SMILES)	4357/797 (PDBbind 2019)	0.84	0.98	15.4
DLSCORE, Hassan, et al. [16]	3191/300 (PDBbind 2016)	0.82	1.15	8.6
KDEEP, Jiménez et al. [15]	3767/290 (PDBbind 2016)	0.82	1.27	7.1
DeepAtom, Li et al. [14]	3390/377 (PDBbind 2016)	0.81	1.31	10
DeepAtom, Li et al. [13]	9363/1000 (PDBbind 2016+MOAD)	0.83	1.23	9.6

**Table 3:** Search space used for various parameters during parameter optimization.

Parameters	Search Space
Filters	128, 256, 512, 1024
Dropouts	0.2, 0.3, 0.4, 0.5
L2-Regularizer	0.01, 0.001, 0.0001
Padding	‘valid’, ‘same’
Number of layers in CNN-1 & CNN-2	1,2

## Data Availability

The data presented in this study are available on request from the corresponding author.

## Supplementary Materials

The following supporting information can be downloaded at: https://www.mdpi.com/article/10.3390/ijms232213912/s1.

## References

[1] Ballester P.J., Mitchell J.B.O. (2010). A Machine Learning Approach to Predicting Protein-Ligand Binding Affinity with Applications to Molecular Docking. Bioinformatics.

[2] Kitchen D.B., Decornez H., Furr J.R., Bajorath J. (2004). Docking and scoring in virtual screening for drug discovery: Methods and applications. Nat. Rev. Drug Discov..

[3] Leach A.R., Shoichet B.K., Peishoff C.E. (2006). Prediction of Protein-Ligand Interactions. Docking and Scoring: Successes and Gaps. J. Med. Chem..

[4] Moitessier N., Englebienne P., Lee D., Lawandi J., Corbeil A.C. (2008). Towards the development of universal, fast and highly accurate docking/scoring methods: A long way to go. Br. J. Pharmacol..

[5] Huang N., Kalyanaraman C., Bernacki K., Jacobson M.P. (2006). Molecular mechanics methods for predicting protein-ligand binding. Phys. Chem. Chem. Phys..

[6] Guvench O., MacKerell A.D. (2009). Computational evaluation of protein-small molecule binding. Curr. Opin. Struct. Biol..

[7] Irwin J. (2008). Community benchmarks for virtual screening. J. Comput. Aided Mol. Des..

[8] Zilian D., Sotriffer C.A. (2013). SFCscoreRF: A Random Forest-Based Scoring Function for Improved Affinity Prediction of Protein-Ligand Complexes. J. Chem. Inf. Model..

[9] Li H., Leung K.-S., Wong M.-H., Ballester P.J. (2015). Improving AutoDock Vina Using Random Forest: The Growing Accuracy of Binding Affinity Prediction by the Effective Exploitation of Larger Data Sets. Mol. Inform..

[10] Lin T.-Y., RoyChowdhury A., Maji S. (2017). Bilinear CNNs for Fine-Grained Visual Recognition. arXiv.

[11] Deng L., Platt J. (2014). Ensemble Deep Learning for Speech Recognition. https://www.microsoft.com/en-us/research/publication/ensemble-deep-learning-for-speech-recognition/.

[12] Pereira J.C., Caffarena E.R., dos Santos C.N. (2016). Boosting Docking-Based Virtual Screening with Deep Learning. J. Chem. Inf. Model..

[13] Ahmed A., Mam B., Sowdhamini R. (2021). DEELIG: A Deep Learning Approach to Predict Protein-Ligand Binding Affinity. Bioinf. Biol. Insights.

[14] Li Y., Rezaei M.A., Li C., Li X., Wu D. (2019). DeepAtom: A Framework for Protein-Ligand Binding Affinity Prediction. arXiv.

[15] Jiménez J., Škalič M., Martínez-Rosell G., De Fabritiis G. (2018). KDEEP: Protein–Ligand Absolute Binding Affinity Prediction via 3D-Convolutional Neural Networks. J. Chem. Inf. Model..

[16] Hassan M., Mogollon D.C., Fuentes O., Sirimulla S. (2018). DLSCORE: A Deep Learning Model for Predicting Protein-Ligand Binding Affinities. https://chemrxiv.org/engage/chemrxiv/article-details/60c73dd4567dfefb56ec370b.

[17] Öztürk H., Özgür A., Ozkirimli E. (2018). DeepDTA: Deep Drug–Target Binding Affinity Prediction. Bioinformatics.

[18] Rube H.T., Rastogi C., Feng S., Kribelbauer J.F., Li A., Becerra B., Melo L.A.N., Do B.V., Li X., Adam H.H. (2022). Prediction of protein-ligand binding affinity from sequencing data with interpretable machine learning. Nat. Biotechnol..

[19] Wallach I., Dzamba M., Heifets A. (2015). Atomnet: A deep convolutional neural network for bioactivity prediction in structure-based drug discovery. arXiv.

[20] Skalic M., Martnez-Rosell G., Jiménez J., De Fabritiis G. (2019). Playmolecule bindscope: Large scale cnn-based virtual screening on the web. Bioinformatics.

[21] Feinberg E.N., Sur D., Wu Z., Husic B.E., Mai H., Li Y., Sun S., Yang J., Ramsundar B., Pande V.S. (2018). Potentialnet for molecular property prediction. ACS Cent. Sci..

[22] Li S., Zhou J., Xu T., Huang L., Wang F., Xiong H., Huang W., Dou D., Xiong H. Structure-aware interactive graph neural networks for the prediction of protein-ligand binding affinity. Proceedings of the 27th ACM SIGKDD Conference on Knowledge Discovery & Data Mining.

[23] Lim J., Ryu S., Park K., Choe Y.J., Ham J., Kim W.Y. (2019). Predicting drug–target interaction using a novel graph neural network with 3d structure-embedded graph representation. J. Chem. Inf. Model..

[24] Cang Z., Wei G.-W. (2017). Topologynet: Topology based deep convolutional and multi-task neural networks for biomolecular property predictions. PLoS Comput. Biol..

[25] Son J., Kim D. (2021). Development of a graph convolutional neural network model for efficient prediction of protein-ligand binding affinities. PLoS ONE.

[26] Stepniewska-Dziubinska M.M., Zielenkiewicz P., Siedlecki P. (2018). Development and evaluation of a deep learning model for protein–ligand binding affinity prediction. Bioinformatics.

[27] Nguyen D.D., Gao K., Wang M., Wei G.-W. (2020). Mathdl: Mathematical deep learning for d3r grand challenge 4. J. Comput. Aided Mol. Des..

[28] Zhang H., Liao L., Saravanan K.M., Yin P., Wei Y. (2019). Deepbindrg: A deep learning based method for estimating effective protein–ligand affinity. PeerJ.

[29] Issa N.T., Stathias V., Schürer S., Dakshanamurthy S. (2021). Machine and deep learning approaches for cancer drug repurposing. Semin. Cancer Biol..

[30] Zheng L., Fan J., Mu Y. (2019). Onionnet: A multiple-layer intermolecular-contact-based convolutional neural network for protein–ligand binding affinity prediction. ACS Omega.

[31] Durrant J.D., McCammon J.A. (2011). BINANA: A Novel Algorithm for Ligand-Binding Characterization. J. Mol. Graph. Model..

[32] Limbu S., Zakka C., Dakshanamurthy S. (2021). Predicting Environmental Chemical Toxicity Using a New Hybrid Deep Machine Learning Method.

[33] Limbu S., Dakshanamurthy S. (2021). Predicting Environmental Chemical Carcinogenicity using a Hybrid Machine-Learning Approach. bioRxiv.

[34] Wang R., Fang X., Lu Y., Wang S. (2004). The PDBbind Database: Collection of Binding Affinities for Protein–Ligand Complexes with Known Three-Dimensional Structures. J. Med. Chem..

[35] Morris G.M., Huey R., Lindstrom W., Sanner M.F., Belew R.K., Goodsell D.S., Olson A.J. (2009). AutoDock4 and AutoDockTools4: Automated Docking with Selective Receptor Flexibility. J. Comput. Chem..

