# Supplementary material for: A New Hybrid Neural Network Deep Learning Method for Protein–Ligand Binding Affinity Prediction and De Novo Drug Design
Source: Int J Mol Sci. 2022 Nov 11;23(22):13912. doi: 10.3390/ijms232213912 (PMC9693376; doi:10.3390/ijms232213912)
Supplement: Supplementary file 1 [file ijms-23-13912-s001.zip › ijms-1943738-supplementary.pdf]

1. PDBIDs for K_d + K_i dataset

1a. Training Set for K_d + K_i dataset:

1o0n	5i2e	3slz	2ole	2jkp	4xir	2vpn	2x7t	5ew0	3nw3	5ovr	3jyr	3kgq
1gvx	2pql	4ruz	1avn	5fl5	5ia4	2xj2	2brm	3kr8	1sqt	3s54	1mai	1o5g
4tun	3vbd	8cpa	3iww	1jmg	1izh	4u8w	2wyf	1n46	5op4	4rqv	3qfz	3dd8
1ajx	5kqx	6c7w	1o3f	2cn0	3fvh	1c86	5bry	3ddf	4o2b	2wej	5vd1	3t85
4u69	4d7b	5om2	2afx	2zz2	4xtz	5jxq	4cg8	3ccz	4r5b	4djy	3d8w	3cke
2gss	4k6i	5ix0	6c0s	3nkk	2azr	5tpx	3f5l	5g17	3ps1	4qew	1v2n	5e3a
4q7w	5i9y	5vd0	5nvv	2oi2	4llx	1od8	5ia5	4zzx	1x39	2h21	6c7x	3zps
3w5n	1wvj	1f57	1e3g	4g0z	2qbq	4dmw	2wn9	2wer	2f1g	4q81	5txy	4ehz
1o86	1sqo	2x00	2qwd	3x00	2aoe	2pwg	4r59	5n34	6eqv	5mqe	3q71	3gvb
4llp	4b6o	2hmv	4aji	3mmf	4knn	2uwl	1c70	5lsh	6gw4	2oxd	1m2r	5vb5
5bv3	4daf	1oau	5wp5	4rfc	3t84	2o8h	2whp	4ih3	4wrb	3oyq	4hwp	5za7
4gq4	4y3y	5ceq	1qbn	3bgz	1px4	4lyw	5htz	6mj7	3g1v	2xjx	2bo4	1k1i
3s0b	5vsj	3b2q	4fsl	3nee	6mu3	4ih7	1b1h	1lgt	2ptz	3k5x	5ivc	4ra1
3p9l	5amg	2rio	2yge	1hpv	4nxu	4ago	3gvu	1ppi	4fs4	4dkr	2qi1	4a4v
3g31	4i54	5l2s	4x8o	4ffs	6cbg	3hl5	2cet	3ehx	6hlx	1dl7	4rwj	4d3h
3qxt	1pa9	2arm	4cg9	2ces	3zt3	1fkh	4rvr	4z0q	1gx8	6fo5	3tf6	3f8e
5ta2	5nxg	2x7u	4egk	2b1i	3iub	4cpz	3axz	3exe	3f78	4o2c	3gy4	4kn0
2xyt	1lyx	1utj	5jzi	1v0k	2vvc	5ka7	4kyk	3fed	2qu6	3f33	6hrq	4ucc
5fou	1fh9	6fuh	3suv	4avj	3rlr	4muv	5isz	6g9i	3oyw	5kej	1b8n	5ie1
5efh	4nku	6hai	2nt7	5lud	1os0	6chp	1lan	2ews	3sk2	2jjb	1ew8	5l3a
6g0z	5ia1	5ipj	4up5	3k97	2q38	3uo4	4o09	3ozt	3mi2	4ew2	2jf4	2zb1
1srg	4nh8	1zdp	2zfs	5gof	2p4j	2qnp	2r0z	4jxs	6epy	2x09	4wt2	6mub
4agq	3cj4	5bwc	5dgw	4wk1	4ibi	5k0h	2wyg	1if8	4o9v	5fnt	2qbr	2p2a
3ibl	4ayp	3uxl	1b3l	5acy	1j37	3qw5	3a6t	3b3s	3pe2	1cbx	3t1m	5e6o
3b7j	2a5s	4jn2	5epn	3uil	5f1x	5flq	1q5k	4zx0	1d4l	1m1b	3g0e	5bs4
4je7	6fhk	3nu9	1g4o	2wor	5nk4	3k4q	3gqz	5w1e	3g1d	3zsx	4bi7	4whs
4tkb	2pwd	2c94	4q93	2p3b	5uov	1ypj	2cbv	4myd	5cbr	5vsf	1drj	5ahw
5ewy	4msn	3o9i	3znr	4zip	6csr	4ht0	4kyh	3ikd	1i2s	4k9y	6fng	1xh4
1zhy	1m0q	3cow	5lvd	3qaa	5oa6	5d3x	2qmg	1ec3	2cf9	4alx	5ect	4qgi
5f08	5fsn	1xff	5eek	5gja	6cdp	5dhu	1u1w	4yo8	6dai	6h2z	1jvu	4k0o
4uma	6gl8	1vyg	6b4n	2xg9	1wm1	6epz	5fto	1lvu	5m5d	4abe	3kiv	1qbv
2fzk	4m2r	2cf8	2yfx	3aho	2z1w	5odx	2aj8	6ckr	3kdd	1c4u	2yz3	2xbv
5anv	1pzi	2jh5	1nvs	1fkf	1v7a	1b6k	4h3j	3h30	5ovp	2pow	4agm	2h4k
6csp	3wmc	1s39	3rm9	3n2v	5i7y	6bbx	456c	3el1	2uy5	2e7f	5ko5	4jne
5u0y	1y3v	2zft	3cft	4ipi	1okl	1k1o	4cwo	2pvj	4e6q	5ot9	3wjw	3mzc
2fxv	4dju	3aid	1hbv	6en5	2i3h	2h15	3lzz	5etj	2csn	1drk	3b3x	2tpi
1v2u	2f7i	1k6p	4ij1	3mam	5mwy	6h2t	4mr6	1f0t	2w4x	3s6t	2v3u	4ngp
3d6o	1y3x	2xab	4w9i	3oy8	1cet	4o3c	3nq9	4fzj	4k7o	5eqp	3ibi	2r9x
3i5z	3r4p	3ekv	1uwf	3zv7	2vw5	5t19	4aci	1y3n	6gji	3juk	1gai	3c88
3pn1	2ccc	6hke	4dkq	3isj	1fkn	3vha	2yek	5wbo	1yfz	3daz	4aba	4re2
5eh5	3mof	2jdu	1qf2	2pq9	1jev	3ebh	4knj	3d0e	3ge7	2gsu	1iiq	6fmj
4p58	2ce9	5neb	5j41	1ezq	5ey0	3ffg	2uwp	5a6x	2fqt	2qtn	1bxo	5e1s
1q8u	5o9p	2c3i	1g7q	2hhn	5yj8	5lwd	3fwv	5nxo	4cgi	3ttm	4ad3	6bm5
2vw1	1p1q	1i9n	2zwz	4exs	3gnw	2uxi	5i3v	1c5s	1x8j	3ip6	5op5	1i9p
3tcg	5itp	2uwd	4xt2	4jsz	5g57	4ca7	5u49	1om1	1km3	4luz	5h1t	3cj2
4gql	3d50	3d1x	2bvs	3qt6	4k4j	5tuo	4yk0	3fx6	2ylc	4zx4	4ibk	4jyb
5vo1	1oba	4n9c	3bex	1a69	4bt5	5dqf	5xvg	4nwc	5llc	2qbp	4cpr	2reg
4uof	3a2o	4w9h	1oif	5cqu	5vc4	2v54	3t0d	3dp9	4uja	4zls	1b2h	2xjj
2v2q	1utm	4kz4	3ozp	1ur9	6g3v	4mhy	4azc	3d51	1k27	1pb8	6f20	3m6r
2pu2	5hwv	1zoe	3e3c	4gfm	6g98	4ybk	1bma	6cjv	2y7z	5j7q	3r16	1bzj
3brn	1ydb	3c56	3E+12	5laq	2afw	6fs0	4hf4	2psu	3n8k	4lxd	5e2l	5ty9
5eqe	1kuk	4z2b	1o2r	5jhb	5hvt	5mgk	3cf8	1v1m	5k1d	2jh6	1rnm	1m2x
6b97	4y5d	6std	4elg	3drf	1ft7	2e94	2zn7	2vqt	4yxi	3m1k	5boj	2oxn
2j75	4jzi	5t7s	5n93	1q8w	3lea	2wkz	5yas	4y79	4r3w	4j45	4css	3f3e
4j28	3evd	1mrx	3ivx	3k1j	6dh7	6gnr	2wec	4e7r	4agn	2d3z	2uz9	6h1u
5dbm	4uc5	1fm9	3vhk	4qnb	2zy1	1e1v	4c52	1x38	2xda	4ek9	2bfq	4or4
3sv2	3iss	6cpw	2clh	5wlo	2iko	4i3z	5oei	2nsl	1q7a	4hj2	4del	4p5z
1ajp	3buf	1ork	5llg	5irr	1gvw	6ghh	3rwp	1y20	1olu	5t8o	4u6z	3iw6
1v2s	2w5g	2vzr	5ipc	3twp	3f68	4ido	6f05	7upj	3dc3	3n4b	3ppm	5nea
5hz6	5lwm	5wa9	1h46	5nkh	1oar	3zdv	5kab	4i9h	5l9g	3o75	4z0k	3ryv
3buh	3djk	4nvp	6eqw	2xmy	5oh4	4mo8	4i9u	2boh	3prs	1xjd	2vnt	1ie9
3bva	6guk	5cbs	5cxa	5en3	1gj6	1nz7	5caq	1pxo	4ca5	4ht2	5wbm	3m3x
3bgb	4er2	4ogj	2yfe	5x62	4nkt	3bgc	3tza	1m0n	1nl9	2bes	5gs9	4lko
4bcm	1hpo	1nhu	1tni	1e4h	4q90	2f9k	4nuc	3t64	2p7z	3uw5	3lp4	1bn4
4na9	1fcz	6g2m	3s77	3ebi	4zow	1xq0	4owm	5f1r	6faa	2hb1	1eld	1szd
1fd0	4afg	5ndf	1yds	4psb	2yk1	4wop	1w3l	2vo4	5fsx	3owj	4zv2	3zi0
5j2x	3iph	6d5e	1ex8	2xyf	5th4	3w37	6htg	5lny	3gsm	5fnf	4asd	4qy3
4ddh	4hwo	1k6t	4ca8	4qjx	1a99	5fho	4pb2	3pcj	3mv0	4p6w	1j17	5j1r
4j3l	1f5l	4lhv	5dlx	2yki	1pgp	5nee	1fzo	1a4r	4az5	1upf	3muz	3pd8
4fcq	4yth	1usi	1qk3	5n3y	1qft	4kao	1yet	5os5	1sw2	3el4	2a5b	3v51
5o2d	4q87	2qpu	5u0d	3i9g	2vl4	2b4l	3p3g	5e2s	1w9u	3rf5	1afk	2c1p
4eoh	3hzk	4erf	5l9l	1ec1	2gvj	5m7s	1nhz	2cle	6d50	6ayq	5mg2	3g2z
2xye	1dmp	4abg	4kcx	2bq7	2xyd	5sz6	2pou	2xxx	2o4r	4m0e	3uz5	3nex
1uml	2gst	1qyg	3s45	6h36	3o4k	5mo8	2aod	1fv0	5etb	3wz7	1bdq	1z1h
1nw7	2fxu	2r5a	1uho	5nn6	3tao	4hym	5u0z	2qta	6czc	4rd3	2yb0	5we9
1f73	4ezz	6b1k	2pyn	4mr3	6f90	3m37	5f2u	2wly	2fle	1fzm	2wyj	2jew
5hu9	3ao4	3nim	2brb	1x1z	6f9v	2yel	4q19	1elr	3vx3	5j64	4ua8	4yzu
4de1	5mme	4jsa	3c39	4qjw	1xh5	1jeu	4aje	1hyo	5d47	5ot8	2vot	4av5
3wgg	5cep	6faf	1e6q	1mq6	3rlp	5f2p	1dar	3k02	5l7g	1rd4	5yfs	5xg5
5otr	5o58	1o5a	1fkw	5g4m	3iw5	3hkq	2qrl	4djp	3a1d	5yz2	2a4m	2w8w
5i7x	3fl5	2xm2	4des	1bnt	2q8m	2zz1	1d4h	2vmc	3i25	4ygf	3aqt	3mdz
5voj	3n86	3fv2	2zc9	5g2g	6h34	3imc	3hcm	1stc	1ebz	1o2h	2oax	1zvx
5nw2	3b66	4mc1	3p9m	5tbe	1c84	4uin	3zk6	4agp	4w9l	1u1b	5ufc	4ynl
3aaq	2hnx	1k1y	1hn4	2zx7	4w9c	2vkm	4ovf	5j27	2vpe	3lpl	1f0s	1v2j
3b92	3jup	5vi6	4gzw	4rfr	3g0i	3kyq	1jn4	2exm	5uf0	2olb	4k55	1o2o
3uxd	1p1o	5fsc	1zpa	2al5	3b7u	1utn	5ijr	6eya	3d6p	4lm0	5fng	4agl

5vm0	1lee	2vh6	2x2r	3c2f	4i7p	1c5t	3hzv	3tzm	1gpn	1ivp	2f7o	5wgp
4pnu	3bfu	3hww	5nkc	4omk	3f34	3w07	2haw	4x5q	5mrp	5ehw	3mxe	2qd6
4djq	4zgk	3e92	5nki	4xy8	4kz3	6cpa	5c2h	3p4v	5ld8	5cs3	5e8f	1ui0
1hih	4ibg	1mq5	5zc5	1lag	5ep7	3lxe	5org	3ekr	5nya	2qe4	4q7p	6gnp
6e4a	6gjj	1a9q	5evz	5cu4	3d7z	3pww	3p8p	1sbg	2v2v	4cwr	4g95	1j4r
4pvy	3dx3	3tif	2wbg	6f3b	2pog	4yhm	2avs	3fzy	5amd	1k1n	4nj9	3ao5
5hjq	3kgu	3f6g	3pwd	4xaq	3k5v	2h3e	4gbd	1j16	2pqz	1hvj	4mn3	2fxs
4pp5	4r5t	3d91	2oc2	4ayq	3rz7	3pb8	4loo	4hw3	1b7h	4kwo	2i19	5oa2
5yyf	5tcj	5eng	3t0b	3tay	5nwe	4mnp	4hdp	3si4	2avo	5w44	5ayt	4r73
1hii	3si3	1li3	6fba	3rbu	4tz2	4qj0	1t31	2pk5	4i8z	5h85	3wto	4jym
1mu8	4gfo	5m25	3bft	1w3j	5d3l	2pym	1sdu	5kr2	1g45	1tsy	1nh0	2r1y
4c9x	5za8	4xmb	6elp	4auj	2isw	1ciz	5d2r	4qer	5eh8	1azm	5m7u	5i3w
3nuo	2r9w	1fzk	6gjm	1m0b	4x6m	1grp	2vvv	1egh	4zek	3lpp	4m2w	2h6b
4iwz	4bj8	2x6x	5ev8	1r5y	4hzm	2v58	3b27	3sw8	4i7k	5er2	1jaq	6equ
3nxq	5wyx	3cdb	6gvz	1bnn	2wc4	4k7n	1u71	1cnw	3eqr	1dhj	2f6t	4w9o
3fv3	3ckb	5mby	4cga	3dyo	1wur	3hkn	4g0p	2uy3	5exm	4gqq	4kfq	4c6u
3qbc	5mkr	4bf6	1c87	5wxh	6gl9	1ai5	2xnb	3coy	4agc	3rlq	2bqv	3rf4
3r6u	3drg	2xn5	4r75	1ppl	1h6h	5el9	5n99	1yqy	5l7e	3e6y	5kat	3u6h
4qfl	3hvi	3gi5	2qtg	4or6	3eax	4cpt	3ppq	6cks	4gj2	3hzm	3myq	1qbt
3pe1	5l4i	1mfd	2pvk	3n3g	2vwo	3arq	4rd0	6h8s	1wuq	3dzt	4u6w	5wqc
3qx5	1hsh	3i6o	1alw	3uug	1a4k	5u0e	3d0b	4kxn	3zbx	4rr6	3ozg	3su5
3f3d	4ufh	4b34	3t08	3gy7	2fqy	4l4z	1lpz	6f92	4d4d	4muf	3o9a	5b5f
4umb	1fao	5uxf	4rlw	5dq8	1ksn	6hmg	5d0r	4loy	5jfu	2ans	3wz6	4itp
4f7v	4er1	3hb4	5fbi	4z84	2xd9	3mhl	1no6	3d4y	3qx9	1b46	3fzn	5nlk
4wn5	3m67	1fjs	5u8c	3hkw	5yh8	1iih	5cs6	4nbn	1igb	5nw7	4q4s	2psv
5mes	5hva	4qac	4rlt	3b50	5sxm	3djo	5i3x	4ibj	5ovc	3b67	4wa9	1dqn
4xiq	4j47	1dhi	1q8t	5os4	5ovv	2f7p	1fh7	1h2k	5l4m	6eij	3s76	4q4q
1uj5	1qhc	3ivg	6d5j	2vrj	5i2f	4xit	6epa	3ttp	1mmr	4ks4	2gzl	1tom
2qwe	2f80	1bgq	3upk	2wq5	3qwc	2qm9	3rr4	2jfz	6fuj	2idw	5qal	3kgp
5er1	6g2l	2qwc	6ey9	1oz0	2weg	4m8h	1mrs	3l4z	1jmf	2ojj	3bl1	1pph
5upz	6gzd	3uex	2qi5	4io4	6ayo	4ahs	3f1a	4hy1	4aoi	1xap	4kow	2euk
2qpq	1o33	2aog	2qg0	4u6c	5ia0	4mgd	1k6c	1hlk	6hpw	3lpi	3v3q	2qnq
2o4l	3tfu	2g94	3bgq	5n0d	3dx2	3rux	1xd0	3n7o	3zns	5fs5	2wm0	2xj7
1phw	2bal	3eb1	3k2f	4m3p	2y80	4oma	1dgm	1sr7	1nfx	5vcy	5j1x	3cd5
1qxk	4b35	1aid	3c8b	3s71	2za0	1nfw	4xu3	1bnv	4zb6	3qqs	3sur	4yho
5am6	3iae	1aj7	3kek	1hi5	6f1n	4ty6	2avq	1nli	5uc4	5i9x	5yjm	4phu
4cu8	1pkx	4lkq	4cig	1tjp	6iiu	4non	1lgw	3p3r	3lxk	5ewa	2wr8	5f63
5var	4aqh	6dh8	4mc6	2bak	3f18	4gid	1f8d	4kax	4x5y	3m35	4y59	5j20
2xej	2wc3	5tb6	3nuj	5hz5	6ftp	5iwg	2vwl	4ty7	2b07	5ldm	6d56	4mrg
1br6	4acc	4f9w	1pvn	4f1l	4jia	1w9v	3agl	1cny	1l8g	3mna	3gjw	3u10
5dey	3zsq	3pbb	6g3a	4av4	4det	3jzj	3rtf	2v57	2wlz	1z71	2ra6	5k0m
3ddg	4u0f	4bb9	3ejr	2p3i	5ha1	4tt2	5zkc	4da5	188l	2nmz	4y0a	3cyz
4k18	4emf	1p19	3hp9	2wca	2ves	3jzh	1msm	6ftz	2xc0	3gk1	6fmc	3u6i
5n9r	5dh4	1ps3	3r4n	5edc	2q1q	4og4	3n9r	4dld	5fl6	3ckz	5g5v	3cj5
1bhx	3bkk	4og3	4jyt	4oeu	4mdn	2j7e	2std	3uev	3gss	2xn3	1w0z	4fm8
4xtx	3d8z	3h5b	5u12	3nu5	4c1u	5ime	2p16	3wvm	4zb8	6dj5	1hps	1bcd
4pg9	3n2p	1ai7	1nt1	6gnm	3tk2	4rpo	1d2e	3o9d	1b6h	5orh	4lxz	5nk7
3jvs	2f94	4leq	1b51	3u5l	3n3j	6d5g	4f6u	1xt8	4ujb	1fkg	5lvl	1hpx
1ii5	2zdl	5mge	3uzj	4cpy	3ap4	5tfx	2j47	2nnd	4pvx	3miy	3vhc	4qge
4n07	4yes	4io5	5wcm	5fns	1elb	4g8n	3gkz	1x8d	3fh7	4qem	3c2u	4ncn
1b5j	3lzu	4do5	1vzq	2nn7	1kc7	4rux	4a95	4cws	4elf	4p6c	5hbs	3hfb
2yxj	4gqp	4cd0	4bcs	2wb5	3qfd	3wtj	1lkk	2rfh	1o7o	6bm6	2vnp	1bq4
5n84	1eoc	4uj2	2q89	6eir	4djo	4fev	5sz5	4mss	2q63	6h38	3t01	4z07
5nkg	1xbo	1yda	3pb9	4oc2	3h1x	3b7i	4zw6	3mfv	1q65	2xj1	1nw4	4iuo
1o36	6eeo	1fki	1pz5	1b58	3veh	4zx3	4l9i	1xow	1v2r	5d1r	3th9	5mn1
4elh	6edr	5ngz	4q8y	3le9	5osl	4unp	5lyn	1rp7	1y1z	4kb9	4u5s	3juo
3zyf	3uod	3f5j	3zyu	2ya7	4lkk	5kcb	5dpx	4m7j	4dzy	2jgs	4ea2	3vtr
5fso	1xka	3qfy	4cp7	4m0r	2ayr	3zm9	5tkk	4jz1	3ewj	4w9p	1hvk	4avh
3k8q	1h4w	1g46	1hmt	2gyi	6hsh	3vw1	2wk6	3jy0	3kv2	3ryj	1qin	2zx6
1gi1	1hms	4zbf	1c3x	1f4e	4o61	3a5y	2xei	1ols	1ogd	1h22	4n6g	3ed0
1laf	4avi	1cnx	3v78	3zi8	3b1m	1amk	4l2l	2f34	4gzx	5ct2	4o07	3k4d
3dd0	3ok9	6cze	1mes	4dst	5os2	4ql1	2xdl	2on6	6e7j	3oe5	5d3n	4ew3
1jyq	2pk6	4wkp	3ga5	5gmh	1c5y	1jys	6elo	5vcw	2hzy	4ibc	4arb	6g35
6b4l	6eif	3neo	4qlk	4ayu	1gno	1o2z	4arw	4q8x	6csq	5u6j	5fnu	1b05
6gf9	1hmr	5j0d	4fz3	4oks	4e4n	1hi4	3roc	1sqa	6aqs	4e67	1owh	1hos
1i7z	2r38	3lk8	1wcq	5ikb	3ui7	3s8o	1k21	6eqp	5ia2	5c3p	2vjx	4y8x
3i3b	5zo8	2o0u	5aba	5wuk	2xxr	5n25	2xii	1ql7	2pu1	2jdp	5n1s	6ekq
1fl3	5hrx	4y4j	5ito	5yft	2ot1	4rrf	3qdd	6h37	4cpw	3u92	5cso	1pzp
4epy	2jkh	2vhj	3a1e	4dff	6d5h	1bjv	1b4z	1e2l	1rtf	1jqy	3alt	3b24
1swr	1tkb	2uyn	5j8u	1m7y	2gh9	4ufk	4a4q	4flp	3czv	6gjl	1ele	4std
1txr	1ebw	3fcq	4gih	3nsn	1gpk	2yfa	5vkc	4ahu	2j78	3ta0	1ydk	4nxv
4qfp	2fgu	4mrw	3kdm	3igp	3nx7	3ts4	1sdv	2ha3	3rlb	2oi0	3tkw	3f70
1b55	3u93	6fhq	3c4h	2v00	5h1u	5fog	3vje	5j6l	4np3	1fzj	1zoh	1qbu
3u8l	1pb9	6msy	2pwr	5jg1	1njs	4g8v	4b9z	4je8	2jdm	2cc7	4duh	4ag8
1f74	1o35	5y8y	2wl0	5uln	1o5e	3bqc	3r1v	1pyn	1xr9	4cjr	5j3l	2drc
5dnu	5fe6	3v2p	2j4g	5aml	6fa4	2w26	2y7i	5c28	3nzk	1ejn	1n4k	4zzd
5mz8	5n3v	3pcg	3sue	3coz	3lp7	4zec	1siv	1o0m	4b7p	3u8n	5hrw	5d3h
1swg	3gs6	5tkj	2nsj	3gm0	6ge7	1bm7	4bcp	2i6b	2cli	2hxm	5iyy	3gx0
4j21	5nkb	5o5a	1gjc	2qd8	5eyr	5d26	2j7f	2zxg	6hly	1xkk	4eu0	3f5k
5f25	4zeb	5kad	4lar	3i4y	5ta4	3hk1	4fxq	1str	3vh9	2byr	3fvn	3bxe
1lpk	4jwk	2oiq	4mjp	4io6	1u33	2zxd	2aoc	4cst	4oc3	3qps	3acl	4bcn
4x6o	2oym	1hvr	2xp7	4owv	3l0v	1tlp	3k00	4llk	3ov1	5fpk	4k3n	2jiw
6eed	1b3g	4rlu	1g2o	4q9o	4bi6	1d3d	5jhk	5k8s	1kpm	1tng	5nvx	4ceb
5ehq	5ekm	6g9u	2qi0	4re4	1z4o	5nxw	2vk2	1t4v	2xdx	5kbe	3t3u	5nw0
6b96	3rm4	2x4z	1bv9	4ibf	1rql	1e2k	3ljz	3acw	5e2p	1kav	1b40	3pwk
5x54	4k0y	4y3j	6gjr	6eq8	1bwb	5dqe	4ivd	4j7d	2ydw	3dx4	5eh7	6d9x
5mmg	1x8t	3kku	3h78	3pb7	5fl4	4x48	1qy1	1p57	4omc	5g5f	2ydt	2v88
3oy0	3hky	3ucj	1o0f	5g61	3iqu	4avs	5nvw	2r59	4ef6	2f35	4wov	4z1e
2j62	3f16	4zl4	6upj	5oha	2x8z	3ubd	3n76	5cbm	6eux	2p3c	3f3c	6g38

1kdk	2pwc	5flt	2gv7	1drv	4uac	3vjc	186l	4tmk	5evb	4m8e	5oh9	5zag
5alb	4z93	4g4p	5avf	4rj8	5c2o	3el5	2pyy	1qb9	1zfq	3npc	1kv5	3jrs
3zll	4iic	3gta	3k99	4o0x	6hd6	3lpk	4msa	2q8h	3ff3	2hl4	1ydr	6cbf
5aan	1fhd	4kxb	4a7i	1lah	1pot	4xya	5g45	187l	3be9	5ih9	4bao	4o04
2fqo	3suf	5tcy	3sut	1sv3	5dwr	5c8n	3d9z	5lz5	4lhm	2i4v	2hnc	4b2i
5ka1	3fhb	4xxh	4u71	2fmb	1njc	3jdw	1jak	4qf7	1uw6	4ymq	3ejp	5oq8
5ioz	5ovx	3r5t	1ela	5d3j	5otc	4cc5	5vc3	4dhl	3umq	4uye	3spf	4xo8
5eij	4crl	1hxb	1lcp	5my8	5o1h	4ndu	4der	3mho	1b8y	5llp	1n51	2boj
1lpg	2p4y	4gzp	4i7m	5g60	4tkh	3ibu	1pme	5n18	3rv4	2yay	5v0n	4i74
4mme	2qrk	2p4s	1pdz	4lch	3gc4	1h23	3l3n	4pin	5sz2	2fdp	5kma	2d1n
4aj4	5m9w	1ocq	2rd6	185l	4k77	4qsv	2pqb	6gon	4b9k	2r3t	5vl2	1kzn
4pow	6guh	3lka	1hvi	6b7b	5jgi	4etz	1hi3	2i4d	3s73	4oiv	1adl	3st5
4czs	2vfk	1b3f	3gcs	4w52	2za5	5y94	4n6z	3kqr	1d6w	2cbu	5vyy	5exw
2yi7	2sim	3f48	4crf	5nkd	2rkm	5fhm	6fnj	1ax0	4y2q	6g14	2zdn	5edd
2fqw	3lq2	4o9w	4qtl	5ja0	4qfo	1sl3	4ad2	3s78	5u4b	1g1d	5eu1	1xgi
1k1j	5nn5	5lz7	4uyf	3mjl	1uvt	5nwi	1igj	4ezx	6b98	5ork	1jgl	5izj
2mas	2a8g	3iob	6b59	5hvs	1uz4	4abh	1pfu	4ly9	6ei5	4km2	4xar	4kqp
1f3e	3gbb	4ly1	5nka	3kwa	5dqc	2ypo	1j01	5e2r	3arw	2py4	1hp5	3m40
5f0f	4idn	2nn1	1sgu	3u9q	5jfp	1pbq	2fzc	3h89	1nm6	4e1k	5i1q	5cjf
1y3p	5ma7	3pfp	3ms9	1lrh	3pgl	2e9u	3p8n	4gr3	4a4w	1e66	4ej8	4m2u
5kh3	2erz	3ru1	2vwn	3udd	1d9i	4b5w	4b6r	3ryy	4e9u	2g5u	1dy4	1g36
3m5e	4zw8	4ejl	3i73	3t3c	4q83	5epl	1uou	4dbm	5i80	5n6s	5dgu	2xef
1ctu	3nhi	4jss	1j36	4np2	3nyx	3exh	1a94	5f74	5uv2	2o4z	4-Oct	5gsa
4poj	1qxl	3a9i	3v4t	2r3w	6b5q	5sz3	5izf	3nik	4att	4fht	1ew9	4m2v
4ghi	1thz	4nra	1det	4m13	5y12	3pwm	1ohr	4jpy	4fm7	3mhm	4en4	5vcz
1atl	5wa8	1aaq	4g0y	2hkf	5fol	3p8z	4zyf	2qbw	3ibn	5cst	3i60	1eby
4uoh	2usn	4igt	3djv	4oc1	5byi	5lle	6gwr	4aq6	4ymb	1c1u	3cd7	3bvb
4ddm	5eei	3djp	1syh	5er4	4k7i	2c97	4b6p	2cht	6fnr	4ufj	4ovg	4euo
3ggu	4nnr	2x96	1qb1	2ogy	4efs	3vhd	2e92	5b5g	1o3d	3s8n	4nl1	5tt3
4ts1	1ftm	5eqy	4tkj	2bys	5umy	4a6s	3td4	5anu	2wvt	2p3a	5o1d	5z7j
5nzf	2q6f	2r2m	3hl7	1d4p	2ovv	3f37	4bck	1bzc	4p5d	4mmm	5cy9	4zv1
5ttw	6ecz	4xmr	3bbf	6gfz	5evk	4h3f	5ost	4q08	2weo	5l7h	1z95	5d0c
5i9z	4k5p	4rfd	3ohi	1b8o	2q54	5lif	1c1r	4gkh	2w47	5m17	3pn4	5dxt
4f6w	1dif	5o9q	1jzs	2q5k	5bw4	3pyy	3gba	3i51	3tfn	3mhi	4gue	2ymd
2hzl	3dnd	4llj	2v25	4tim	2tmn	6h33	3sm2	4yc0	3pcf	5j82	1kyv	2o4j
1ajn	3bu1	4rfm	4p6x	5ef7	5oss	5l8c	1g98	4j7e	2d0k	1v1j	4ymx	4xtw
2qbu	1fh8	2j7b	5os7	2jds	6dj2	1b57	6czb	3u8k	4kzq	2bz6	5c2a	4iif
2f2h	4ase	5dit	4x8u	1ik4	4u5n	3oaf	3u5j	4rpn	6fgg	1mtr	2v59	3mi3
1rpf	6ezq	5oh3	4ih5	4gu9	5f1h	1pro	4ax9	6cvf	5msb	5l9o	4lm2	2vvs
4b1j	1ghz	1s38	1t7j	6fv4	2pvu	4rww	1koj	5dkn	3gst	5lyr	5qa8	3b3c
6f1j	1h0a	5e73	5ji8	5nzn	3qox	4abd	3s43	3mf5	3c8a	4u43	1bju	3b4p
7std	1v2w	6eqx	4iie	5j6m	5vd2	2dri	1g7f	2zdk	2wnc	3dcc	2xbw	3t5u
1ro6	4ha5	1atr	3wtn	2rkg	2ccb	4ieh	4o05	3m3z	5his	2fpz	3pju	2qhy
6mjf	4x24	6f9u	2wzm	3iof	3arp	4wiv	5nz4	1f0u	5f1v	3dk1	5fnc	4cwn
5fh8	4f09	3ppr	3oim	4djw	3o84	4c5d	4mmp	4m6u	5fyx	5y13	5sz0	1a4w
4ysl	1flr	5o07	4oc5	4qgd	3ml2	4bqg	2bza	3tz0	3ip9	6ebe	3liw	4n7u
4jal	5ldp	1met	4hu1	5a81	4bak	3q7q	1amw	3ryx	3iue	1ndv	1w4q	5z7b
4i8n	5hwu	5xpi	4tqn	1ua4	3vd4	5k03	1lf2	3sfg	2gv6	3ljg	3qgw	4dew
3su3	4rqk	3huc	5ueu	5j6n	3f19	3q44	4io7	1elc	2epn	2h4n	2j79	3ml5
2bt9	5twj	1i1e	4h81	3ai8	3rv8	3r4m	4ryd	1zog	1ghv	4e0x	2vh0	4jfs
3ekt	4b8y	1ajq	5u0w	3d1y	4n7m	4aia	3m3c	2qci	6hqy	2izl	3rdq	2vb8
4q4o	3pgu	1np0	2zfp	5vgy	2uxz	5yhg	4l6t	1hfs	2q64	5hbn	3zqe	4djr
2uyq	2yaz	4gg7	3upv	5ewk	1k9s	5gmn	4oak	3uri	3uj9	2zjw	2ra0	4ipj
3ta1	3o99	3fvl	4db7	6fe1	5d21	4cr5	1cps	6ayr	5vih	5oh2	5azf	5j86
4app	4mo4	1w5v	1oyq	4a6b	3ebo	3zlr	5zaf	4pp3	4dsu	4pv5	4b7j	5xva
5l9i	5nxp	1mu6	4ksy	3bxh	2vw2	1m4h	6fyz	3l3m	3b25	1fq5	4b3c	1ghw
4km0	3o5x	1yq7	1w11	3old	4lps	3s0d	3gi4	3g34	2qzr	1tq4	2qw1	4p3h
5llo	3qqa	3ioc	5e74	1bxq	2f81	6eln	4ih6	4q46	4kwf	4q1x	5h9r	1wn6
3udh	1e3v	2rkd	2b7d	1mue	1y6q	4i71	3i7e	3ebp	1g30	1b6j	2w67	1uwu
2ya8	3tvc	2a5c	2rke	3gc5	2bet	4cr9	5otz	3pd9	4ykj	4yb5	4qf8	4ciw
5ljq	3g32	5d25	3m36	3r17	4tte	5hcv	4ezr	3ppp	5egu	3lvw	1kui	5d45
5edl	3ckp	4ck3	2i2c	3hku	5jsq	1o30	2ha2	1b0h	4zzz	4mq6	4lzs	5dus
1w7g	4o3f	1k1l	1epo	6eog	1nki	1t32	2rkf	5ufr	3g2y	5khm	6g34	1n1m
5d24	2r23	2ypi	3bra	4hfp	1v11	1eb2	1d4k	5l30	2vuk	4kmz	1mmq	4cwf
4lj5	3tfp	1dud	5ka9	5e13	5jxn	6eq1	3hub	3pck	3cfn	4q7v	6hh3	4und
1c5q	4h75	6ma2	4fnn	4e70	2vvu	6b4u	2hmu	5vr8	5vb6	1vyf	5i88	5mrb
5b2d	5g1a	4r76	2v7a	5ota	4efk	5ouh	2q55	1z6s	3arx	3kr4	1ctt	4cps
4k3h	3t82	4cwt	4q3u	4jfm	1njd	5v82	4loh	1w5y	1oyt	4btk	4cj4	1usk
1m7d	4deu	5u13	1a1e	4cwp	5n0e	5vij	4loi	4q09	4jkw	2p53	2p7g	6dak
4oag	3t1a	4w97	6dil	4lbu	3sr4	4ibe	3c52	3p3t	4de2	1moq	6g39	3uuo
3l4x	4yrd	6f9g	1ec0	6dh2	5ult	3ozr	2doo	5ula	2z4o	4b3b	2wnj	5hi7
4qfn	3cct	2zym	5nk2	2pvm	4l19	1k4h	3v5t	1xh9	4pp0	3zdg	3fee	6gfs
1ttm	5hrv	1g2k	5owl	6got	2weh	5j8m	4in9	3tu7	4dfg	2zcr	1ikt	4b3d
4ngn	1m2p	3ouj	1qka	4qpd	4q1w	5duw	4kwg	4lov	1bzy	1ai4	5cc2	4zbi
3cs7	5g5z	6h29	2vwm	4azi	4kz7	2pbw	1v2k	5meh	1nvr	3l59	3c89	2j34
1ws4	4do4	6ma3	2we3	2gj5	1c1v	1bai	4ytc	4n5d	4q6e	4hws	4bah	1y0l
2ctc	1ype	2cbz	5cau	2qg2	4jpx	4asj	3uxk	4g5f	5cqt	2q2a	5g2b	1o3l
2r43	1erb	1rnt	6d2o	1o2q	3uew	4xas	3r24	3su6	1w5x	4jh0	1bn1	3bwj
3sug	1fcy	1w13	1m7i	3kmx	6eiz	4h85	1kjr	1e5j	1sln	4u1b	4zae	3l4u
6ayi	5qay	3nq3	4q7s	3cd0	4cl6	1jet	5vd3	5u14	6fui	3t83	4tln	3tsk
3u8j	3d1z	1c5o	5x74	3ctt	6dj1	4mhz	5t8p	1lhu	4kp5	4abb	1gwv	2i80
1rjk	4mc2	6gla	2c92	4hge	2d1o	5tbm	3v7x	5mek	1gfy	5j9x	4eor	1v2l
4zo5	4ad6	4j44	2p15	1zsf	6el5	1m5w	5cks	4iva	5i3y	2ez7	1f0r	2qbs
3nu4	5ivy	4cwq	5mjn	1iy7	1nny	3ioe	3d52	1nfu	6exj	4isu	5vja	5orj
2ha6	5oot	3zze	5een	1jcx	3t8v	1t7d	4lrr	1z6e	3dp4	3p17	2baj	5mpz
4h7q	3iod	6b4d	5std	4dko	4keq	2yix	1n0s	2w8y	1onz	1bcu	2hjb	1o1s
4wko	2j7g	5mod	2v2h	2r58	2gvv	3fj7	4m12	5evd	4lm1	1gar	2iwx	2b9a
5orv	5kqy	1ez9	4r4c	4jyc	1v48	5nk6	2zq2	3i4b	5upj	5wex	3po6	6fnf
6e9a	3dbu	2yhw	3pcn	3s5y	1hsl	4twp	6glb	5za9	2j4i	4b7r	3o7u	4pcs

4lm3	2zx8	1xws	4wkn	3sxf	3cz1	4x8v	4kni	3lgs	6fs1	3o9p	3vw2	1nje
4fai	5n17	3g0w	1ppk	4pqa	3s75	5efj	6ced	6fe0	4fxp	5n24	1zp8	4l51
1s5z	1zgi	1g48	2pvh	4qpl	1a9m	5ftg	5z99	4qev	3s9e	5d3p	2xhm	5h8e
2y82	2aac	4jx9	2cgf	3tt4	3fur	5o4f	6ma4	3d83	2pv1	1o2j	1o5r	1utl
1s89	5hcy	1yei	2q88	4ymh	4zwx	1w3k	4o0y	3kgt	2wos	1lzq	2ri9	2hb3
4us3	4v24	2fu8	3r88	3dne	1bwa	1zc9	1m83	5t9u	3su1	1ta6	1nc1	3d4z
2j95	3f80	1hwr	2w8j	4qdk	2vmf	2j2u	3fuz	3p5o	184l	5g4n	6eis	2xdk
1s19	1b5h	5jsg	2wed	4zwz	2cej	4f39	2web	6dj7	2hah	6eyb	4ks1	3gtc
6dh6	1d09	1o2w	2x97	3p5l	3su0	6g36	1o0h	3k8o	4xip	3v2q	3el9	6ex1
5jop	5d6j	4gkm	4ual	2wvz	2xm1	4ivc	5lne	5o9y	1nq7	1e6s	3vf5	2oag
3ozj	4m14	4bup	4bc5	3wtm	4u54	3bzf	1nja	4ara	1odi	4hdf	5n2z	6eiq
1qbq	4s1g	4xty	4b73	2xjg	5edb	2yme	3ahn	5dex	4ynb	2wzs	4pf5	5gj9
4x5z	4ahr	5mwh	4f3k	3ni5	1ydt	1lbk	1ucn	5am7	3rz5	4q6d	1r1h	1hxw
4cfl	1nw5	4e4l	5ose	2ya6	1mjj	4q4r	4nbl	3u90	3vf7	1c88	5efa	2v95
6f28	3n9s	1g85	2i0a	1uto	4c1t	3gcp	1o3j	2jxr	5g46	2weq	1b32	5v7a
3hv8	4o0a	4pmm	3mz6	3ebl	5dh5	1c5p	4az6	3rz0	5htl	2r75	3zhx	4dkp
4j22	4g8m	4e6d	3m96	4ge1	4g0q	4ury	5tuz	4o0b	3bxg	4rn4	4cjp	1j14
3sus	4eb8	1v16	3hmp	5xmx	5jy3	3ao2	1ndz	2gz2	3r7o	2p09	5aoi	1fo0
5jss	3qtv	4q9y	5oh7	3aas	3shc	5e89	1uz1	1n3i	4g8y	4ko8	3t2q	5lvq
4b32	4rsk	5ave	2qnn	2v2c	3cda	4b2l	5m23	2xde	3po1	5mnn	6ep4	5lsg
2q8z	1m0o	1r1j	1d7i	3ocz	1qkt	1apv	6bdy	2c3l	1c5c	5om3	3f7h	2xht
4gj3	1afl	4kz6	5mpn	4ibb	3e5u	3cm2	2aqu	4baq	5csp	1oxr	2qd7	1k6v
3q2j	4eo6	1tmn	1g35	4mpn	5fhn	1cgl	4i7l	4v27	4rhx	3uu1	1qbs	5elw
4d1j	6hh5	3tb6	2wtv	3zt2	2bfr	3gcu	5umx	1d6v	4b5s	4o2p	5fe9	4a6l
5wal	1f4f	2vk6	2xeg	3oil	3zso	2xib	5btv	5o1f	4pft	3gt9	5nbw	4i72
4zw7	1rr6	5ut6	1hvs	3mfw	5d48	5fot	3sjf	3hl8	1qf1	4ca6	4xu0	3t6b
4u0w	2zcq	3elc	3n1c	4iue	5km9	4gu6	2fzg	4rd6	6cvv	4uj1	4nja	5njz
4dy6	1ws1	1zea	6css	4b5d	1gi7	3b26	5hz9	4fp1	3djx	1r9l	2qtt	6guc
6fhu	1kug	4pop	3jya	6fnq	4urz	1uz8	5h5f	3fat	1kmy	3rsx	5mxf	3rz8
2b1g	1sb1	1d3p	2cbj	3n2u	4m0y	4b6s	2qi3	1bty	4csd	2qi4	2w08	3qto
4ipn	4c1y	1fcx	3uyr	4ap7	3nes	5i8g	1zge	5uez	3hf8	4u70	3fas	3bgs
4f3c	3fv1	1k1m	5ivv	6gg4	3nox	3d78	5ljt	5ucj	1a28	3l4v	4lm4	1gi4
3v5p	3ipu	3l4w	2o4n	3nht	1nfy	3ifl	4umc	2qdt	6gue	5fdi	2r2w	5lso
5flo	6dq4	4b0b	2e27	3wha	2wjg	3t0x	3p3s	3s0e	3fuc	2xxt	6cwn	1bnq
6ht1	5d3c	1ceb	4ll3	4zcs	5dyo	1g7v	3dgo	4lj8	3v2n	3qxv	2jg0	2pcp
3ocp	4xoe	1fch	5jq5	4ggz	1if7	2vc9	5f2r	3myg	1mh5	4cu7	2p7a	2o4k
4f5y	4fys	3o56	3ies	4nyf	4xoc	5wl0	2fgv	3hu3	6ma5	3d7k	6cfc	4io2
6cdo	3kjd	4o6w	5oxk	1lbf	4rdn	5a7b	4fl2	5h8g	2vvn	3f6e	2e2r	5mrm
4kp8	6ghj	6rnt	5jt9	3bbb	5ey4	6cwh	4r74	5u28	1dzk	5ov8	4rrg	3ivc
5m77	3q6z	4buq	4cs9	4fl1	3g19	5n0f	5vcv	3m8u	5j6a	1df8	1ado	3ng4
1nf8	1fkb	5yl2	5f9b	1gaf	5ne5	3p7i	4qij	1lst	3ip5	4emr	2hu6	4u5o
5ant	4eo8	4io3	4ewn	4gny	4bks	3ug2	5llh	5nap	5dw2	5jvi	1fzq	4hbm
1jlr	5fh7	3rdo	3re4	5n1z	4zzy	1duv	1g52	4tpw	2j7h	4tu4	3f17	2x0y
5upf	1q91	3eko	3mhw	5hct	3hvj	4cmo	6h5x	5b25	1a30	2bok	2xog	4o97
4r5a	2i4x	3e93	3k8c	5sz7	3ag9	4ykk	2ax9	2w66	6cdl	2wky	3g5k	2cer
2ojg	2p95	3bv9	4sga	1ssq	3nyd	3cl0	2bvr	4dv8	1ec9	4ngm	3aau	1yvm
4iid	3hit	1v0l	3cyw	1d4y	1f8c	3qgy	1i5r	4gki	3b4f	3ip8	3gdt	2x95
2qwb	5sz4	4tmn	2i4u	2am4	5tmn	2vsl	1bxr	3k37	3b5r	3g30	6exs	4gr0
2i4w	4yml	5nxi	4fk6	2zq0	4m0f	1kzk	2j94	4qf9	4kx8	5tp0	1ecq	1nwl
3o9e	2xb8	4bs0	3h8b	5vb7	4bny	1fiv	1pr5	5cj6	6c7q	1ndw	1i37	10gs
2zcs	3dx1	3ikg	2qwf	5z5f	3p58	3ewc	4f2w	5kby	1mfi	1usn	6cdj	2vo5
5uoo	3f7g	3utu	4lvt	2z94	1h5v	2hoc	4m8x	3b65	1t5f	3vdb	1lyb	4wkb
3oe4	4q99	2xpk	1izi	2v77	2e91	1add	1qji	4e3g	1c5x	3hig	5mlj	5exl
1sh9	2ceq	4pee	6dif	2zmm	4kiu	5drr	3suu	4ufl	4j48	4msc	3pce	4zw5
2y5h	3u81	3egt	4ish	1nc3	4gzt	3hll	4ufm	2jh0	1y6r	4qd6	3std	3f7i
5c1m	2xb7	3g35										

1b. Test Set for K_d+K_i dataset:

4cjq	5xo7	4rra	3lzs	5ive	5os8	5uk8	4azg	6gjn	4jfk	5ko1	4l4v	3c79
6ce6	5fut	5jsj	5k9w	3bpc	3n35	5ad1	5m04	4w9d	4ddk	5kly	5fnr	5xs
1p1n	4ozj	5o9r	2nmx	5ufp	5d3t	5wyz	3c2r	1lnm	4ljh	3rz1	5oqu	4b33
4ab9	2ewa	1b52	4lk7	2vpo	2evl	4r4i	3eeb	5u4d	6ey8	2rcn	4mre	4ovh
2vyt	3zln	6eol	5u0g	3f15	4i7j	4trc	4b76	5cp5	5ia3	3ueu	2vwc	5nk3
4np9	4xu2	5oku	1li6	4yyt	5t9w	4yx4	5ltn	1oe8	5lz4	5tef	2vt3	5h1v
4qsu	2j77	2vxn	1b9j	5jox	3o8p	1kel	3qkd	5lom	4j93	4i8x	4hp0	1uv6
1qkb	4xu1	1ugx	5czm	5i3a	3c84	5a5q	5fls	6euw	4pox	5o9o	3g3r	4zba
4q4p	5fov	4gah	3f8f	1qk4	1wdn	3up2	3usx	5u0f	3bug	5nau	3dri	5jgq
4pzv	3ryz	2fw6	2vba	4ocq	1bnw	2fqx	3s8l	2yi0	5mnr	3c2o	3kmy	2clk
4w9j	4dcs	2pqc	5n2t	4f0c	4aq4	4zji	5yhe	1h2t	4xk9	5js3	5dx4	2q7q
4q0k	1rpj	1g54	4x50	5afv	4mul	1bhf	3ery	4de5	2xbp	6bhv	3ovn	1hk4
1bp0	4zvi	3vvy	1ogx	2v8w	2j7d	3w9k	5efc	3zsy	5ehr	6gzm	2cgr	1li2
1lkl	3a1c	4nbk	6g37	5nk9	4b5t	1bn3	1xk5	3fjg	3mss	5ehv	2pgz	5mpk
4azb	3ekx	4pum	5n31	1l83	4d8z	3p2e	5fe7	4qyy	4b74	3ccw	5ya5	1msn
4x5p	4r4t	4axd	1b38	6b7a	4ajl	5c5t	3ldp	1td7	4f9y	4abf	4l50	5kva
4cp5	1r0p	5t9z	5lli	1q72	1w96	3s72	4knm	5m28	1yc1	5mgj	2cex	5nk8
1mfa	4xtv	6ckw	3q6w	5i29	5uff	5eis	4r0a	4zme	5fnd	5ii2	5ulp	2buv
5f60	4ymg	5e7n	4pfu	5chk	4w9f	3kdb	4r4o	3ekw	1ogz	1rbp	1g74	5mro
2zkj	2vmd	5aol	5igm	4poh	3hmo	1rmz	5fck	2c80	5l4j	2amt	3f8c	3ime
3jrx	5orw	1q1g	1odj	1ydd	3ekp	2rin	6d55	5cp9	3ujc	4r06	2i4z	5kax
4bqh	5g4o	4hpi	5nw1	6exi	1yej	5f8y	4x5r	3kdc	5aut	5aqz	2ygf	1b4h
1yc4	4w9k	1b5i	1d7j	3fql	1lke	6eyt	3w9r	2ca8	5mgf	5fsy	2w9h	2r0h
5lvr	2gl0	2wuf	4kn1	5btx	4ibd	3ljo	6gnw	4lzr	6fni	1mrw	3wtl	5ei3
1sld	5fox	1b3h	4qp2	1olx	2wzf	4qxo	5eq1	1qaw	4br3	1bnu	4zt8	4qb3
1f4x	5aoj	5om7	1kv1	4kif	2i4j	4v01	1os5	3bxf	3gy3	5u11	5v79	5f62
6evn	5nyh	3ove	3gy2	4zei	1xpz	4bkt	3qlm	5a2i	4x3k	4q3t	4omj	4tjz
5tmp	1m48	2wf5	3da9	3wzn	3ujd	3tmk	3dsz	2rcb	5ihh	3d2e	4ai5	1g53
6q3q	1qy2	3ijh	2zdm	5tya	5f5z	5f61	3t2w	2bmk	5nze	1uwt	3sww	6ej2
1gnm	1bv7	1g2l	1g7g	5vp9	3own	5ufs	4c2v	4i5c	3ipq	3vd9	1h1s	3su4

2wgj	2fx6	1pxp	2uwo	4ivb	3mhc	4ei4	1loq	3fqe	4mc9	1n5r	4rak	1yp9
3oku	2y8c	1f5k	4u73	3vfa	2bvd	1f4g	3eft	3n7a	5aa9	1jsv	1jq8	3hs4
4cd5	1o5c	1b6l	5sz1	1hvl	1zs0	5g1z	5e28	1k22	3djq	4riv	1d4i	4j46
4qll	4z83	2pov	1n4h	3sio	1w5w	2vj8	4z1j	4gr8	5j7w	1ql9	5c1w	2qhz
2fvd	4f9u	1efy	1wc1	3t09	3gv9	2qi7	3zpu	3hkt	4h42	4ufi	5mks	3ejq
1ec2	5fcz	2hs1	1o38	1v2o	4bam	4djx	5n1r	1trd	1x8r	1ajv	5a6k	4clj
4h3g	3vfb	1g3d	2h4g	5l8a	1enu	4oc0	2f8g	5hz8	3wz8	4hla	4x6n	2zda
5sym	3gi6	2bpy	4bf1	3iog	2y5g	1qbr	3jvr	3gr2	3t60	1owe	1xhy	1my4
5mwp	1vso	1yqj	2qi6	4mrz	1qan	1vfn	4cd4	3l4y	2oxx	2xbx	1q54	1qbo
5egm	1ecv	3ldq	1hdq	2zgx	5exn	1oss	1z9y	1tcx	4ou3	2ihq	5k1f	4bqs
6cn5	1m2q	1jao	4z1k	4kzu	1nvq	4nh7	4heg	1ppc	2nta	3fvk	2dw7	3suw
1mrn	3ehy	3acx	5ny3	1e1x	4bt4	1gyy	1gyx	2y81	4djv	4q1y	1sdt	2glp
1tpw	4cra	4n8q	5zae	4i8w	4crc	2v3d	1w4o	3bkl	3zc5	1xk9	5kr1	1ndy
5upe	2x91	1ppm	4zx1	4yha	1syi	2avm	2ewb	4dq2	4nue	5l8y	2e2p	5e2k
4few	2r5p	3uw4	1f8e	2gkl	1c83	4a6c	6g3q	4gqr	1qf0	2iuz	4qrh	966c
3b68	5e2o	4dsy	3p8o	2j27	1tnh	1u0g	3dln	1hee	1o2n	6dh1	5cas	5fdc
5kz0	3gbe	3e5a	3bl0	1ypg	1ony	5j8z	3q1x	5kr0	5cap	4gii	3t70	6dar
1xug	3zcl	2oxy	3l3l	8a3h	1o3i	1gnn	2cen	1wht	3zxz	2a14	3nb5	4crb
4de0	4riu	6evr	3hek	1g3e	3b7r	3str	4bt3	2pvl	3mxd	3lir	3o5n	3zq9
1w4p	4bco	1ogg	3kmc	2flr	4n9a	3n0n	2bpv	3zdh	5zaj	1z9g	3su2	1k4g
1pxn	3zj6	5nih	3nu3	2y5f	1tx7	4hdb	2uy0	2br1	2xys	1d4j	2y7x	2i3i
4ban	1v2t	1c5n	2rk8	2jke	4ruy	1ghy	2h6t	1hvh	3s2v	3ozs	2rka	2ovy
3nu6	2e1w	1f8b	3lmk	5dfp	2ihj	2d3u	1fpc	3cyx	3ffp	1q84	1qb6	4e5w
4m8y	2xc4	1g32	4isi	2uy4	5ti0	6ej3	3rt8	3b3w	3sha	4g90		

2. PDBIDs for K_d dataset

2a. Training Set for K_d dataset:

5vb5	3uod	2fxs	4o9w	1elr	2cgf	4k9y	5ha1	3f48	3vha	5fs5	3v2p	3cj4
4ceb	6cvv	2hnx	1kjr	5jop	4n7u	3p5o	3axz	4w52	1ftm	5lz5	2wvt	4efk
4w9l	3c2f	3ivg	3dp9	5u0w	3zqe	3hww	5nw2	6ftp	5lvl	5mpn	3lka	4aq6
1lag	5tcy	1fcx	3f70	4u5s	2xib	4w9c	4ybk	2wer	3ryx	4und	4n6g	5j27
4yk0	6ma2	5l3a	4fxp	4mo4	4ly1	4dbm	3s0b	3s78	5exw	2jdm	1qkt	4uc5
3rlp	3ppp	2r58	4ido	4av5	3uev	4qac	5ovc	3ryv	5g46	3m6r	3wgg	3dzt
5mes	2rio	3pwm	4y2q	3e5u	2vmf	2wej	5izf	4ibk	5d0r	5n84	5dus	4ayu
3kmx	4arw	2ogy	5ut6	1oau	1utm	5ia5	3tkw	2a5b	4l9i	3wto	5x74	2w08
5aba	1v11	2g5u	3f16	4up5	4q46	4b32	1if8	5j0d	4ymx	4ury	5wbo	1xr9
2wc4	4je8	4c1u	4yxi	2xn3	5mod	3vje	5yh8	3r24	6g9i	2pqb	1ttm	3pgu
2gzl	5ie1	2v57	1bn4	4dsu	3upk	1fzo	1xap	5vyy	1oz0	5d24	5vm0	1e3g
5edl	5km9	2xp7	3kr8	3fat	5lsh	5cxa	6h2t	2a4m	6ma4	1b6h	3n9s	5l9i
3f1a	1e3v	4mrg	5x62	4ad6	2vk2	5m17	3jyr	5eqe	5cqu	4og4	4cjp	4xu3
2jf4	4wt2	4nkt	5mge	4kcx	2q88	5ovp	2h6b	6g0z	4i3z	4y59	5fe9	4y4j
3jup	4aba	4r75	5g4n	5wbm	3p8p	1onz	4pvy	6guc	5fnf	1nw5	4ysl	5wp5
3ms9	3ru1	1b46	1ax0	1lkk	3qqa	5osl	5e2l	3mho	5ucj	1k1m	4abd	5etb
5i3x	5fpk	2drc	3rz5	4io6	4y3j	4yrd	5ftg	5umx	5var	5ma7	4j3l	3isj
4css	1b4z	5ufc	2hzl	2bfr	4mnp	5boj	4tun	3u90	6gji	2e7f	4ad3	3ebl
2wq5	4cst	3ppq	5cso	1qy1	2zq2	4az5	4loh	5l2s	4ibb	5anu	4urz	6ckr
1b5j	2haw	2xj1	2j78	5g1a	6g39	4jwk	6fhk	1fo0	1bn1	5aoi	5u0e	2fgu
4oag	1qk3	5yyf	4u54	5o1f	4jym	1sw2	5vc3	5duw	4qj0	1d7i	4r4c	4k3h
4jkw	5h5f	1bnn	4ymh	4b7p	4ffs	3drf	2reg	2w8w	5n3v	1ogd	1k1i	5nkg
1olu	2w47	5hrx	2d0k	4dkp	4ara	6fba	5mrp	3rsx	5tpx	5llo	3cow	4ghi
6htg	4n07	4k4j	4gzx	5k0m	3v78	5j82	2p7g	6g36	4qfo	4dew	4jfm	6f20
5fbi	5i1q	3x00	4hj2	2v00	3buh	5meh	6mjf	2hjb	4egk	5eh7	4ezx	5mxf
5i9y	1k1n	6gon	3alt	4dkr	3mhi	3vx3	5fot	1s19	4zb8	3hky	2xii	5ka1
4rvr	1y3p	4u43	3arw	4ibi	2p3i	5wyx	5lwm	3r6u	5i3v	4aqh	4q9o	3hub
3lp7	5o9q	6faa	3r1v	5nz4	1xh4	4qgd	3nhi	3ouj	5hjq	2bak	4np3	1hms
4pop	5kma	2vzr	2zdk	3ed0	6fa4	5am6	5nkd	1oxr	2b9a	4xu0	3ioc	3nq9
3nes	3a6t	3npc	2xjg	3ioe	5u14	5ja0	1kdk	5ect	1njc	3b26	5j1r	1n0s
1xow	5njz	2qm9	3bzf	5fsn	1a9q	4l4z	2yix	6g14	1jmf	5c2o	5cs6	4duh
5edd	1xh5	3cd7	2hmv	5twj	4u0w	1tq4	4g0p	1g45	3uo4	5xg5	1b32	4m14
3myq	2pyn	3n1c	5kcb	6g34	5txy	6gla	4k7i	1g4o	2wor	2ccc	3lk8	1b1h
2dri	1oar	6hsh	3brn	4og3	5ldp	4qdk	4fxq	4k55	4rpo	3iue	1b51	1li3
4i7k	1q7a	1w3j	5a81	2c3i	3gy4	3lzz	6csq	1d4p	5egu	4ibg	4ht2	6eux
4l6t	4der	1pgp	2byr	1ucn	6glb	1ie9	3udd	4i9h	6ghh	3bxg	2xj2	4abe
4d3h	1m7y	4qfl	2c97	2v3u	5evz	6el5	3u10	3zt2	4pnu	4xt2	4epy	2clh
4g0y	6elo	5g2g	6gjm	4n7m	5h85	3w07	5l4i	3umq	4mmm	4qpl	5j20	2uyq
3vhd	2zwz	4agn	5d25	1q8t	1xws	4b1j	6gjj	4lbu	4q4s	4j21	3wtn	6gnp
4cj4	5jsg	3s71	2we3	5u12	4pp3	1i7z	4luz	3b24	5msb	4rwj	3cfn	1p1o
3p9m	1i9p	1g48	3rm9	4arb	2qbu	3e6y	5zo8	4nja	2x4z	3b25	6csr	2zdl
5oei	2cli	5mwh	4azi	3tz0	5d3p	5d1r	4cjr	4bqg	5ji8	4qer	3q71	4idn
4or4	1lbk	4wn5	4zek	1k1j	5ih9	6fhq	3m3c	5lle	5f1h	4f7v	5irr	2j79
5ia1	4emf	4etz	4np2	4lyw	4agm	5d3n	4pf5	2h3e	5hva	4gq4	4w9i	5orv
3cz1	3hzv	6g38	3mam	6g98	5lz7	5org	5wa9	1okl	4xya	1m7i	4aje	1aj7
5E+74	5vd1	1uho	5mek	6gw4	3bbf	2tpi	3nik	2c92	1fkf	5lif	3zlr	2v95
3r7o	5l9l	4r73	3ip6	2yel	2whp	4ezr	3kgt	5f08	5lny	4qf8	6fs1	2xn5
2zb1	5ikb	1k6p	1lhu	3m67	4g4p	1ql7	3mhl	4gqp	4det	4whs	1a99	1nki
5nvx	4q08	3k1j	2cbz	5k8s	1drk	3udh	4oiv	3gcu	5n9r	4ddm	2yk1	4uac
4rqv	3zso	5i88	6gwr	2iko	2pyy	2v59	5i9z	4jal	5ia4	4ih6	1g1d	5ia0
2zfs	2cbv	3ao5	1jev	4ad2	3vjc	5ewy	4fm7	5vd3	4b34	2uyn	3a1d	5cs3
5j1x	5fe6	5ivc	3gk1	1uwf	2qtn	4b35	1b3f	5aan	4m13	5bw4	4kao	1mai
5y8y	4qsv	4dzy	5fnc	5g4m	4qjw	4lzs	5wuk	1zhy	3elc	6fgg	1rd4	5ost
4ynl	1utj	5vcz	6fnj	6ey9	2wos	2pql	4oks	4qjx	4jyb	6mub	1erb	3coy
5nvw	5bs4	5f2u	2v25	5j6m	5d3h	2ha3	3cyz	3a1e	1fao	4b2l	4dko	6fmj
4ymq	3hkw	1rnt	2oiq	5z7j	2wn9	2xm1	4myd	6eq8	6g3a	2w8j	1ez9	5jss
4muf	1w9u	6f1j	4xtx	5zkc	2yki	5yj8	2x00	4i7p	5ot9	1m83	6ekq	2o4n
3o9p	6fv4	6epy	5u13	6gjr	6hly	3jzh	3ga5	4leq	3dp4	4mpn	3g0e	2nn7
4c5d	2wyf	2q8h	3ryj	3fwv	2vrj	5hrv	6exj	5l4m	4bb9	4loi	4b3c	2boj
5flq	5e89	5t7s	3rz8	1s5z	3qx9	1nw7	2vb8	4psb	2pk5	5alb	2xht	5vl2
4fm8	3cj2	4cga	2gh9	5i2f	3qps	4tqn	5j3l	5g5f	4kxn	4kqp	1q8w	4ayq

3s77	1bzy	1jmg	3gvu	1usi	6b7b	5o1d	3exh	1zea	6bm5	3rz0	3fj7	4qew
4abg	3a5y	2weq	4rqk	186l	4zb6	3zt3	3s0d	3bex	5jzi	4muv	2zdn	5vr8
3lp4	5nkb	1g46	5o5a	5orh	4io4	4oeu	5kbe	3iqu	5i3w	4b3d	5yft	1cnx
5dqe	4w9h	1b3l	1r9l	2xdx	2nnd	1k6t	5mz8	6chp	4a6s	5ovr	2b4l	1utl
4mr6	1nja	4cg8	5vgy	5gja	1rr6	3zyf	3nim	4dy6	1xh9	4x8o	3exe	3zyu
4m7j	5j2x	5csp	1sv3	1d3d	4uin	5my8	5cqt	5yz2	5gsa	5e73	4zv2	4ejl
6h8s	3ekt	6hqy	5nwe	6f9g	4lps	2euk	5cbr	4d1j	4gue	1uz8	5htl	3sk2
1uto	3uj9	4f1l	1dar	1hmr	1xq0	2zft	2fqw	1c5c	1bm7	4k7o	2wnj	1jeu
1v16	1m7d	4ahr	4m0y	3fzy	5i9x	2pym	5izj	4q3u	4tt2	5eh8	4c9x	4rlw
5otc	4hfp	4uoh	4abb	4j22	2xdl	4xtw	4yho	5ork	4nku	3ao2	1bcu	3k8o
185l	4dst	3bxh	4ly9	6fe0	2w4x	4pb2	4o0a	4bi6	4or6	5d21	2cle	3k5v
4rfr	4q6d	6epz	5oha	4l51	5mgk	2vuk	5jgi	4knj	2yb0	4asj	3cft	4ncn
5os4	4km0	4btk	5ia2	5fou	1swg	2olb	5ota	3gcs	2r1y	5ehw	5lsg	3b4p
1b58	3cj5	6fo5	1lgt	3w5n	2yme	5fog	2vw5	4e1k	1jet	4o3f	2qpq	4csd
6cdp	1hn4	6cks	4zzx	3m3x	3juk	1hsl	1ex8	4knn	1njd	1msm	4rd6	4tkh
3f3d	5nkc	4cl6	4kax	5otr	4rlu	5n99	5tkj	1jgl	4avh	5j9x	4uof	4yhm
4xy8	5ovv	4bcs	3o4k	6hpw	2xbv	3ryy	5hwv	4jpy	5mpz	5f63	3zi8	5gs9
5x54	5u0z	2j7f	6guh	4e67	3po1	1yet	3b27	5ekm	4m3p	4ql1	4xty	5gj9
3ov1	5xpi	3b2q	4qtl	3uug	3nht	2q63	2xjj	3m5e	6czc	4i7l	4ksy	3zdv
3rdq	1t7j	2i6b	2weh	3bu1	3pck	1lgw	5isz	5z7b	3u5l	5b2d	4aj4	2v88
1d6v	3zhx	5fyx	5nk4	4ddh	3vw1	3fvh	5ta4	5kad	4qnb	2izl	5oa6	5llg
5e3a	1qyg	5ivv	3juo	1ydk	2vpn	1avn	5n93	1fl3	5gmh	3arq	6cze	4ts1
4rpn	4zyf	1fzm	4z93	5tcj	4j7e	4h75	1usk	4g8n	4pft	3kgu	4ipi	5i80
2ce9	3m96	6bdy	4wop	5llc	3zm9	5oa2	1ikt	4fnn	5xmx	4bj8	3n8k	6gl9
5u0d	5epn	1szd	5tbe	3k8q	4ua8	4ge1	2aac	5iyy	6gnm	6d5h	4ih7	1det
4x5y	4x5z	3s75	3jrs	5ivy	3zsq	2cbu	3zi0	3ozj	3drg	4o2c	1kmy	5ty9
3s73	4x5q	6fyz	3b50	3p3t	5tbm	4q4r	5ldm	4pp0	4jpx	1w9v	5eyr	3cdb
4mq6	6ge7	3pju	4rd0	5ka9	1cnw	4c6u	1gwv	4ogj	4nra	5uxf	5wxh	3kiv
4d7b	1igj	6fnq	5mnn	1sr7	5yhg	2oi2	3o84	1h6h	3fhb	5o58	5vb6	2p15
4y3y	5llp	3el5	6hlx	5ko5	3s76	3pcj	1srg	6gl8	1a1e	5hvt	2yfa	6eog
4a4v	3f78	3vw2	4bi7	5d3j	1cny	5yjm	2ans	6fuj	3zsx	4db7	1b0h	5fsc
6hai	4cc5	4flp	2j7g	5u49	3zk6	4ieh	4nvp	5mn1	4c1t	5n17	5epl	4m8e
5i3y	4q6e	5nk2	3qfd	4fz3	3e12	4nxv	1mjj	3bxe	1u1b	2ylc	3lpl	2doo
5g45	4mme	4crl	6epa	3cda	5vcy	5ey0	6hd6	3hv8	3ubd	3ta0	2xdk	4v27
5ito	4ra1	2q89	5nbw	2nsl	4zeb	5efa	6fui	3f19	3v4t	4xo8	5fns	4qpd
5j8m	4n5d	3imc	4uyf	3nee	3q7q	4zec	1b5h	5l9o	2o4l	2j7b	3zll	4avi
5h8e	5eng	5vcw	4poj	5cst	3gy7	5acy	4oct	2bfq	5ipc	3nw3	1y3n	5ufr
2r0z	5om2	3i9g	2nsj	5i8g	2p7z	3upv	5cks	5ahw	3c4h	5o1h	4h81	5vc4
1odi	4dkq	6hrq	2y7i	2qpu	2wnc	5h1t	3iob	5om3	5nkh	5fng	3v3q	4ipj
4av4	5orj	6csp	4b2i	2pvu	4b5w	4yb5	6bbx	5am7	5gmn	4unp	4cfl	1bnt
5qay	3ekv	2v58	4cgi	4omk	6h1u	3f37	6d5j	4f6u	4qev	1oif	1v1m	4pvx
1pot	5wa8	3k02	5n3y	3s8o	3u5j	2pk6	1fd0	2yek	5g17	5m9w	6gfs	5o9p
2ez7	4k6i	4agp	6mu3	2xjx	6ei5	4att	1n4k	5qa8	5j64	5f1x	6eqp	6czb
5kab	4ndu	4o61	2f34	3rlb	3td4	3gx0	3gcp	2ymd	4eo6	5umy	6gzd	3arp
6hmg	4p3h	3rz7	4kow	5lyr	2h21	4gzw	5jxn	3uex	6ayi	2iwx	4lxz	3huc
3d0b	6fmc	5ewk	4ef6	5h1u	5u0y	3i73	4rww	3uyr	5ka7	4d4d	5his	5nwi
1bgq	6eq1	1qft	4ayp	6b5q	5sxm	4qfp	3lq2	4b73	4fzj	4kzq	3bva	4ucc
1drv	4ht0	1pz5	1ws4	6d56	3arx	1lah	6gnr	4mr3	4iue	4jn2	5ld8	5fh8
5g61	4wiv	3nkk	4auj	3t6b	3h1x	6e4a	3ifl	4nbl	6cvf	3neo	3fzn	5ouh
1o7o	2arm	4del	2r5a	5ipj	5odx	5u4b	5lvd	4q4q	5a6x	1flr	1laf	3wtj
4erf	4ezz	4u0f	5i2e	1str	5nvv	4lkk	4c52	4iuo	3kqr	2pv1	1g85	4ahu
6gvz	6mj7	4rrf	5v7a	2cc7	3d78	1pzi	1yda	3bvb	5cu4	5dkn	4f6w	3aqt
4tkb	4c1y	4ej8	6gjl	6gfz	3ivx	2j7e	5dqf	5lvq	6hke	2xde	5azf	4mn3
3mf5	5y94	4cg9	2uxi	5d3x	4agq	1q8u	6g9u	2r23	1nw4	5jhk	4euo	1dhj
5os7	3oyw	3wtm	5btv	5fnt	2hmu	3bra	4cig	4qf7	4zzz	1atr	4q4o	1ork
5f2p	5dlx	3jvs	2c94	5d26	5mme	5otz	4q09	6f1n	4agl	2weg	4dmw	4kmz
1utn	3p9l	1i9n	2q64	1fzj	3ap4	1t7d	1e4h	5er4	3pyy	2fxv	6cpw	1drj
5nlk	2weo	2ccb	5f1r	3f3e	4abh	4lov	5fsx	6eya	4pow	5b5g	3tcg	5vi6
3v2q	3buf	5f25	3el1	5g5z	4kp5	4jx9	1hmt	2gz2	4z07	5gof	1mrx	3iw5
4io7	5anv	2jdp	5kej	1xt8	3cd0	6d5g	1vyf	1b2h	1fzk	2gj5	4nl1	5tuz
2hkf	4gu6	5c3p	2j75	1fcy	5h9r	5f0f	5hbs	4i54	184l	5eh5	5nka	2py4
4e70	6fng	4pp5	5hbn	4bup	3ccz	2bys	4g5f	3pcn	3p3r	5eqy	6b1k	5n34
5ct2	2baj	3el4	1x8d	1fcz	5mrb	3acl	2f7i	6elp	5nn6	3hmp	4ago	5e6o
5jhb	1kzn	4fcq	3ckb	4j7d	1ii5	2c1p	4wk1	5eu1	3h78	6ezq	5e1s	4pg9
4azc	3fh7	2ha2	4nxu	3ta1	4y5d	4nbn	6msy	2r75	4rr6	4lj5	1vyg	4km2
5dnu	5lyn	2bt9	4daf	2fqy	3miy	1i1e	4jyc	3coz	1swr	5i7x	4pmm	2vpe
4ibe	4zow	3tay	5ep7	2vhj	1d2e	3t1m	5nki	5os2	3tao	5bv3	4hu1	5nk7
5cc2	2a5c	5fnu	5ioz	4tte	5nk6	6d5e	4b3b	5j6l	1b55	1dhi	3hzk	2mas
4zgk	1ydb	4rsk	4ibf	4mmp	3tk2	4xmb	5vd2	4mgd	5itp	5ttw	4p5z	3uil
6ced	4tmk	1k1l	5mqe	6fhu	3ttm	5ave	4h7q	4qem	5ovx	1tjp	4avs	2ces
5lwd	3iub	5e8f	2yay	2nn1	2ews	3ao4	1y0l	5ijr	3cd5	5dbm	4non	1d3p
188l	2vfk	3gm0	4kfq	6bm6	1p1q	4b8y	5hvs	4phu	6gue	4ko8	4czs	5i7y
4uye	4lhv	3tsk	2cet	4rlt	1m5w	5ngz	5ta2	4aci	3o75	187l	6eln	3c39
5fso	5d6j	1b05	5l9g	4igt	4i9u	1jyq	5eqp	4zzd	4w97	4g0z	5vsf	4zl4
2h4n	3r5t	1tsy	1b7h	1uw6	4nnr	3ttp	3lxk	1u1w	3kyq	1b3g	5g60	5fol
5dq8	4zv1	3nq3	6guk	4kn0	3e3c	1gx8	4ih5	3el9	5hrw	5hu9	3lgs	3ip9
5tb6	6eyb	1amw	5oss	2ha6	3s8n	4k7n	1h0a	2v7a	5en3	5os5	2fxu	2q2a
5mmg	4jne	4je7	4ck3	5d3l	2ypo	1zc9	5uc4	4us3	3nex	2bal	3gnw	3ppr
4h85	3f80	4rd3	4m12	5cbs	1lrh	1nli	6g35	2xab	5mg2	2yaz	1lvu	3m40
5o2d	6fuh	2f35	1k6c	3k00	1ols	2ydw	6ftz	1a4r	4ibj	3hcm	6fs0	1adl
2jdu	5mo8	4pin	2jgs	4lar	4avj	4aia	3oy8	3qbc	3nx7	6d50	3gkz	5tkk
3mhm	5vd0	4k0o	5z99	1k6v	4tkj	2vo4	3bwj	2yhw	1qka	3iw6	2a8g	1pfu
3rm4	5o9y	4fht	3tb6	5j86	1oba	3jzj	5flt	3rux	5hwu	3iod	5t9u	2qbw
3uew	4io5	4deu	1g7q	2yi7	3vhc	1mfd	1b40	4gu9	4aji	5k03	4b5s	5n18
3kv2	3f33	1a4k	5lne	4g0q	5nw0	2x6x	2qta	4xtz	3sfg	3lzu	5w1e	2vmc
6ma3	5m5d	4kni	5kat	3tf6	5ot8	5yfs	5kh3	6gf9	3f34	5cy9	1bnv	3hzm
4ahs	2ra6	4i7m	5b5f	5f74	1yei	6fnf	4qfn	2uwd	3qxt	4ipn	3evd	4xmr
1kyv	5lud	5n2z	3l59	5ose	4des	1nje	4n9c	2yge	4nj9	3tif	4m8h	4a4w
5avf	4ibc	6fe1	3f18	1dl7	4u5n	2p09	1p19	2zym	4loo	6css	5qal	3wvm

5j6n	5j8u	4tz2	3cct	4xxh	5d0c	4g8m	4eu0	3kdm	3kdd	3lzs	5flo	6dq4
4b0b	2e27	3wha	2wjg	3c79	3s0e	5jsj	3fuc	5ad1	2xxt	6cwn	1bnq	3rz1
6ht1	2ewa	5d3c	1ceb	4ll3	3zln	5dyo	1g7v	4trc	4b76	3dgo	4lj8	4xu2
3v2n	3qxv	2jg0	2pcp	5ltn	4xoe	5jq5	4ggz	4qsu	1if7	2vc9	3qkd	4i8x
5f2r	3myg	1mh5	4xu1	4cu7	1ugx	2p7a	3c84	2o4k	4f5y	6euw	4fys	3o56
3ies	1wdn	3up2	4nyf	5nau	5wl0	3ryz	2fw6	2fgv	2yi0	3hu3	6ma5	1h2t
6cfc	4xk9	3kjd	4o6w	5oxk	5a7b	4fl2	5h8g	2v8w	2vvn	3w9k	5ehr	3f6e
5nk9	1bn3	2e2r	5mpk	3ekx	4kp8	4pum	3p2e	4b74	6ghj	6rnt	5jt9	3bbb
1b38	6cwh	4r74	5kva	5u28	1r0p	4rrg	6ckw	5i29	4r0a	2buv	5e7n	5m77
3kdb	3q6z	4cs9	4fl1	4poh	5fck	3jrx	3g19	5orw	5n0f	2i4z	3m8u	5j6a
1df8	1ado	3ng4	2ygf	1nf8	1fkb	5yl2	5f9b	1gaf	5btx	3p7i	6fni	1mrw
5ei3	1lst	1bnu	2hu6	4u5o	5ant	4eo8	4ewn	4gny	4bks	3ug2	5llh	5nap
5jvi	1fzq	4hbm	1jlr	5tmp	3rdo	3re4	5n1z	3dsz	4ai5	4zzy	1duv	1g52
4tpw	5nze	3f17	2bmk	4tu4								

2b. Test Set for K_d dataset:

4cjq	5xo7	4rra	5ive	5os8	5lso	5uk8	4azg	6gjn	4jfk	3t0x	5ko1	4l4v
3p3s	6ce6	5fut	5k9w	3bpc	3n35	5m04	4w9d	4ddk	5kly	5fnr	5xsr	1p1n
4ozj	5o9r	2nmx	5ufp	5d3t	5wyz	3c2r	1lnm	4ljh	5oqu	4b33	4ab9	1b52
4lk7	2vpo	2evl	4r4i	3eeb	5u4d	6ey8	2rcn	4mre	4ovh	2vyt	6eol	5u0g
4zcs	3f15	4i7j	5cp5	5ia3	3ueu	2vwc	5nk3	4np9	5oku	1li6	4yyt	5t9w
4yx4	3ocp	1oe8	5lz4	1fch	5tef	2vt3	5h1v	2j77	2vxn	1b9j	5jox	3o8p
1kel	5lom	4j93	4hp0	1uv6	1qkb	5czm	5i3a	5a5q	5fls	4pox	5o9o	3g3r
4zba	4q4p	5fov	4gah	3f8f	1qk4	3usx	5u0f	3bug	4xoc	3dri	5jgq	4pzv
2vba	4ocq	1bnw	2fqx	3s8l	5mnr	3c2o	3kmy	2clk	3d7k	4w9j	4dcs	2pqc
5n2t	4f0c	4aq4	4zji	5yhe	4io2	5js3	5dx4	2q7q	4q0k	6cdo	1rpj	1g54
4x50	5afv	4mul	1bhf	3ery	4de5	2xbp	1lbf	4rdn	6bhv	3ovn	1hk4	1bp0
4zvi	3vvy	1ogx	2j7d	5efc	3zsy	6gzm	2cgr	1li2	1lkl	3a1c	4nbk	6g37
4b5t	1xk5	3fjg	3mss	5ehv	2pgz	5mrm	4azb	5n31	1l83	4d8z	5fe7	4qyy
3ccw	5ya5	1msn	4x5p	4r4t	4axd	5ey4	6b7a	4ajl	5c5t	3ldp	1td7	4f9y
4abf	4l50	4cp5	1dzk	5ov8	5t9z	5lli	3ivc	1q72	1w96	3s72	4knm	5m28
1yc1	5mgj	2cex	5nk8	1mfa	4xtv	3q6w	5uff	5eis	4zme	5fnd	5ii2	5ulp
5f60	4ymg	4pfu	5chk	4w9f	4r4o	3ekw	4buq	1ogz	1rbp	1g74	5mro	2zkj
2vmd	5aol	5igm	3hmo	1rmz	2c80	5l4j	2amt	3f8c	3ime	1q1g	1odj	1ydd
3ekp	2rin	6d55	5cp9	3ujc	5vcv	4r06	5kax	4bqh	5g4o	4hpi	5nw1	6exi
1yej	5f8y	4x5r	3kdc	5aut	5aqz	1b4h	1yc4	4w9k	1b5i	1d7j	3fql	1lke
6eyt	3w9r	2ca8	5mgf	5fsy	5ne5	2w9h	2r0h	5lvr	2gl0	2wuf	4kn1	4ibd
3ljo	6gnw	4lzr	4qij	3wtl	1sld	5fox	1b3h	4qp2	1olx	3ip5	2wzf	4qxo
5eq1	1qaw	4emr	4br3	4zt8	4qb3	1f4x	5aoj	5om7	1kv1	4kif	2i4j	4io3
4v01	1os5	3bxf	3gy3	5u11	5v79	5f62	6evn	5nyh	3ove	3gy2	4zei	1xpz
5dw2	4bkt	3qlm	5a2i	4x3k	4q3t	4omj	4tjz	1m48	2wf5	5fh7	3da9	3wzn
3ujd	3tmk	2rcb	5ihh	3d2e	1g53	6q3q	1qy2	3ijh	2zdm	5tya	5f5z	5f61
3t2w	2j7h											

3. PDBIDs for Ki dataset

3a. Training Set for K_i dataset:

3gba	5nxo	5n1s	4djo	4hf4	3dd0	3s5y	5ljq	1m1b	1qhc	1f5l	4q83	2xxr
5evd	1uou	4u6z	2nmz	2w5g	3g2y	3t64	5n0d	4ovg	3ipu	1qbn	3cs7	1me
3c88	3r92	2bet	6b4d	1e1v	1fkn	2erz	1e6q	1xbo	4r59	2rd6	4kb9	2jh5
3v7x	4b6s	4o0x	1z1h	2qd7	4u70	3ebo	3ikd	1pzp	2vvc	4k77	1dy4	6f9v
4jfs	2wzs	5yas	3uxd	2pog	3p8n	4bt5	1c87	1ai5	4asd	1zog	4xar	4y0a
4gkh	2rkg	4iif	2f7p	4lrr	4dff	2al5	1mrs	3kku	5ehq	4cwn	3u9q	1c1r
2zjw	3fvn	3zps	3p8z	2pvm	4r3w	3dnd	5efj	1d09	3c52	3sxf	2b1i	1bxo
3h5b	4ufh	1bty	3bl1	1gvx	3lpi	2v2h	4hws	4itp	1od8	4bck	3ge7	3n9r
1o3l	4z0k	2r9x	1px4	5dh5	3t85	4cr9	3daz	1w3k	4ty6	3oe5	6gg4	4oc1
4az6	5el9	3hl8	1c84	5mwy	2aod	1mq6	3d1y	3eb1	2d1o	1zgi	4o3c	3t84
4oak	4mc2	2wbg	1o0m	1lpk	1wm1	3ml5	1hih	4elg	2za0	3ebp	1tlp	4o9v
1moq	2j34	3spf	6f05	3ibn	2wl0	1jqy	6dak	3b3c	3v5t	1k27	1ps3	4gkm
2z4o	4rfm	1m0q	2vl4	4ovf	1nfy	1o2w	4z2b	5wex	2q55	4b5d	3d83	3i5z
3dne	1amk	5e2p	4u71	1zfq	3mxe	1pb8	4b6o	1pvn	3u8j	4ase	2j2u	1tni
2xeg	2fzc	4iva	1dif	3b66	1bxq	1uz1	5n6s	1ebw	4wa9	3ozp	1v2u	2uz9
2p2a	3bgz	3wz7	3bft	4tim	4ivc	1hwr	3su1	2azr	3ozg	4umb	2xye	1lzq
2bo4	1o35	5w44	4omc	2qe4	5er2	1m0n	3ohi	3pwk	2fdp	3shc	5evk	5tuo
4q19	1y6q	1mmr	3s54	3uxk	1ciz	4agc	5za7	1pph	4e4l	1k21	1d4h	1v2r
1zge	1qbt	4bao	3n2v	2hzy	3acw	5ayt	1ctu	3vd4	4owm	6ayq	4zae	2rkd
3kwa	1ai7	4o0y	5ew0	1hfs	2gvv	3rlq	1hbv	5ndf	1ro6	4kz6	5vih	1n3i
1jys	1hxw	4oc5	1hvs	1fkw	1qbs	3pd8	4kwf	3k97	1upf	2ctc	5hz9	3wz6
3p4v	4cp7	3kek	3kr4	2p3c	3bkk	4yo8	5edb	5t8o	1zpa	2g94	3r4n	4fev
1lcp	1c3x	4cwr	4ij1	2afw	4e6q	1ppk	4z1e	5fdi	3o9d	5m7u	1ajx	3qto
4cu8	3sue	5amg	1ebz	3nu5	3g0i	4x48	4hym	1a69	1yqy	1w7g	2f9k	3po6
3gsm	2wb5	4cwq	6eis	1thz	2qwc	6eir	1gar	3aid	4mrw	2zc9	2wk6	2gst
2qw1	1oyq	2boh	4cwt	6h2z	2zcr	1lpg	3c8b	2vh0	4q81	4i8z	1z6s	3i60
2q54	4hdf	3k5x	3fv3	2qd8	3qdd	5k0h	4hzm	2xej	6eqv	3znr	3hfb	2hb3
5khm	3f6g	2fzg	6eeo	3k99	3fur	5u8c	4de1	5e13	3r16	1o2h	4cd0	2csn
5elw	4m2v	4cwp	4p6w	2zx8	3fvl	2zn7	3dc3	4m2u	4hwo	3mdz	4a95	2f94
3r17	2hl4	1t32	1c5s	5htz	1phw	2avs	4cpt	1rtf	2f2h	1wuq	4mjp	3fuz
3mjl	5fto	1a9m	2ot1	3m1k	2x09	2o8h	3iof	2jkp	5za9	2ovv	2jds	4zwz
1no6	3ljz	1g7f	3t3u	5fl6	3sus	5oot	5n25	5we9	1tkb	3ozr	3f5l	5c28
4wkn	2w67	4kyh	5l8c	2fle	1qbv	3cf8	6dh7	4g95	5jfu	3gss	4fai	3p5l
6eqx	6e7j	4ca8	2o0u	5hcy	3d0e	5hz5	3d6o	6h33	5n24	3gst	4e7r	5xvg
2pvj	4p6c	1fh9	4do4	5dqc	1hi3	1bcd	5e2s	3iww	3fee	5er1	1ghz	3pfp
3ahn	4nh8	4q1w	4hdp	4keq	5oq8	4oc2	3k4d	3uri	5dgu	1o2z	2p53	4pv5
6h29	3st5	4oma	4l2l	2sim	1xd0	2pwr	5upz	4rfc	1s89	2bvs	3su5	4er1
1apv	1w5y	6eed	4l19	3b7j	3mfw	2zx7	2xbw	4a6b	5dex	2p3b	1ele	3ebh
1ajn	1j14	4gbd	6fnr	2j95	3v5p	2aog	3o7u	1k1o	4zo5	5aml	6cpa	4app

3g2z	2qzr	1hi4	8cpa	4llp	3oy0	1v2s	4exs	2brm	2xd9	1c5o	6eij	1r5y
3ucj	3mfv	1eby	1o86	3ibi	3pww	3mof	2vvv	1g2k	4o09	3t8v	2bqv	2jiw
6g2m	1km3	1kav	1elb	1qin	5mjn	3fed	3roc	4eor	4yc0	4ufj	1e2k	4mhz
1b8n	4djw	1om1	3hk1	2ypi	5oh9	6eqw	6ebe	4djp	5hcv	3t3c	2jkh	3ejr
3ehx	1epo	4ykj	3pcf	4hw3	1alw	1ec1	1bwb	3oyq	4cpz	3uz5	2vnp	3hvi
1rjk	4i71	3su3	4g8v	1j37	2qi5	1elc	4g8y	3pwd	3g0w	2vwn	2pow	1ydt
5oh7	2rfh	3czv	3t08	1fv0	5neb	3gs6	3kgp	4ty7	3rlr	5fhn	2r43	1hii
2xxx	3uu1	2y7z	5eek	4m0r	2wr8	3vf5	4k3n	1lf2	4ea2	4iie	4mc1	4lch
2qd6	1v1j	3qox	4k5p	4gqq	1bma	1ajp	1k9s	3i3b	1o30	1z4o	4w9p	1j16
1f74	3uw5	4p58	1sb1	3f5k	4h3j	2uwl	3f7h	4q90	4a7i	5j7q	6hh5	1m0b
3uzj	1hps	6dj7	3g31	2ydt	2wyg	2zfp	5ev8	2bes	4xit	1b6k	1ghv	5c8n
1dgm	3tu7	3suf	4y79	1ndz	4uj2	5cbm	3vf7	4v24	1eld	3nzk	4zw8	4lkq
5vo1	5th4	4ks4	2fzk	3nyx	2p3a	2on6	1z6e	4yth	4rhx	5vij	2p4s	1qf2
1s38	3a2o	1h22	1k1y	1nl9	1owh	4ha5	5exm	2q38	4bah	1hvj	4gid	4ek9
1sl3	3twp	1gi7	5uf0	6eiq	4x6m	3u92	1br6	3agl	6equ	3sv2	3ai8	3tfp
1fki	1ksn	1uvt	3pe1	1u71	2qbr	3veh	3tfu	4q1x	4gfo	1gi4	4wrb	2qwd
4bcp	4rj8	1cet	4i74	4qlk	3dcc	2zz2	2i4v	1np0	3t01	5y13	1o2r	3pb8
3mz6	1x8j	3dbu	3slz	1y3v	2r2m	5fl4	7upj	5vja	1jak	3c8a	3zze	6ayo
4std	3u6h	3i51	3u8l	1y1z	1o0f	2q8z	3owj	2uwp	6e9a	4kz4	4cpr	1rpf
1xka	5zaf	3rf5	4wov	4lm4	2hah	6ecz	3uxl	3d1z	1o3d	2cbj	3oil	4ynb
5hz6	1ydr	1bzc	1j17	6h37	3gi4	2h4k	2f81	1iih	4z84	2v2v	1sbg	3pn4
3q44	4hwp	3ebi	4cws	4x8u	2q5k	3iss	1ft7	1pyn	5vkc	4dfg	2cf9	5jy3
3qtv	2pwc	2j62	3n86	3mi3	3eax	3b67	1o1s	4rux	3uuo	1f0u	2i2c	4iic
4e4n	2wtv	1hpv	6dh2	1zdp	4ujb	1w4q	1e6s	1eb2	2vjx	3qaa	1ws1	1egh
4ew2	4jz1	1hvi	5g5v	3cke	2ya8	5nzf	3t0d	1fh7	2vqt	3pb7	2f1g	1nt1
1qf1	5wal	2qwe	1aid	1lee	1f0r	1e2l	3fl5	3dyo	6b96	3tvc	5l7g	2aoe
5edc	1c88	4llj	4oc3	6f28	2j47	2qci	3tza	5fhm	3d9z	1f8d	2qi3	1hpo
6dai	6d2o	1c5y	3jdw	2pou	2fpz	2uy3	3hl5	1c70	2f7o	1f57	4pqa	1f0s
3djo	3pd9	2wec	3rtf	2zx6	3nu4	1bv9	4f39	4jss	4i8n	4q7p	1xff	5ef7
2qrk	3b1m	4q93	2b1g	3g34	5e2r	5kqx	1pxo	1p57	3jy0	3dx3	5op4	4gg7
1gfy	4msa	1ela	6upj	4qge	3zns	1nfu	3n7o	1v2l	5caq	4de2	4zls	3s6t
4ivd	3tfn	1fh8	2xmy	1ua4	5m23	6en5	2p4j	1n1m	6h38	2i3h	5u6j	5sz2
5een	2qi1	4o2p	1stc	1w13	5ix0	2uy5	2qg0	2wca	3ddf	3c56	5laq	2f80
3d8w	5wgp	1nny	1qxk	1o3f	4ruz	2vot	1mu8	1aaq	3zv7	4re2	3sw8	3ui7
6b97	5dit	4p5d	2psv	3mmf	1xkk	2r2w	3jya	3su6	1zoe	1bdq	1dqn	5std
1kpm	3bgb	1iy7	1jvu	1w5x	2qtg	6iiu	3fv2	4en4	4q7s	3ml2	1m2r	2xei
3i7e	1gno	4rn4	1mu6	2ves	2pu2	3f68	4j28	1oyt	4zw7	3t0b	3aas	3m35
4mc6	1f4f	1hxb	5nzn	3pgl	1qb9	2oym	5g2b	3wmc	3o9i	1syh	3nxq	1vzq
3r4m	5f1v	1j4r	1ajq	4djq	4a6l	3mna	2vwo	1jaq	4j44	4b7j	3dk1	3bgq
3gjw	4a4q	1nhu	4cpw	6dj5	1nh0	4x8v	1nfw	3i4b	1ui0	1fkh	2yz3	1gi1
1ohr	1fq5	4do5	2hb1	2yxj	5fl5	2aqu	1sdv	3dd8	1jcx	3ggu	6f9u	4f3k
5bry	4iwz	2oi0	4jsz	1kc7	4jxs	4r5t	1bju	3lea	5amd	1njs	3d50	6aqs
4nwc	3mzc	3t1a	1uwu	4cps	1c1u	1o36	3c89	6dh8	5etj	6ayr	2uxz	2r59
4djy	3ni5	3f5j	4j47	3f3c	1ik4	1l8g	3gc4	1nz7	4q9y	4n6z	2gss	2oax
1ocq	4m6u	6b98	5bwc	1gpk	5nea	3ok9	2qdt	5oh2	1pro	3i4y	2gv6	3l3n
1sqt	5op5	5o07	4kz7	1g2o	3u8k	1nvr	6cbf	5t8p	2aj8	1ppl	3t82	3le9
1yds	1o3j	1s39	2v2c	1q65	3w37	2qnq	2e9u	3l4x	2pwg	1pb9	3ljg	4pcs
1ai4	4h3f	1o5g	1qxl	4zx3	1q5k	4yes	4m2r	4q8y	3nuj	3vh9	3b92	2j4i
3o9a	3f8e	2xm2	3q2j	1gj6	1igb	6dh6	3qw5	2wkz	1fm9	3lxe	1ype	4isu
3sjf	1hvk	4xir	3i25	1txr	5jg1	5o4f	3sr4	3si4	1v2w	3prs	2f6t	4f9w
2vvs	4jh0	4u1b	1o2q	1ejn	3bgc	4lko	3suv	5v82	3ctt	2idw	1fkg	4q8x
2ri9	3rf4	2yfe	3su0	2bq7	1c5q	1n46	4f3c	1ivp	3v51	1pme	3dx2	3t83
3ibu	2r9w	4ca5	3old	2fmb	3be9	6f3b	1zsf	2b07	3nox	2o4j	1bjv	3rwp
3b7u	4ymb	3hf8	1os0	5fho	6cjv	3ddg	4o0b	1h4w	3tzm	4uj1	5wcm	4na9
1kv5	3mv0	2zz1	1rp7	4lm1	3mi2	2pqz	2cht	4gql	2vw2	4o07	5j41	2i80
3n2u	6dj2	2q6f	5ljt	1g30	5dgw	4q7v	5nee	4umc	3rv8	6hh3	3fcq	2isw
3k2f	1m4h	3d1x	2avq	3r4p	1j01	4alx	3b7i	4b6r	4mo8	1jzs	2qbp	6got
1b6j	3gtc	3th9	1d6w	2brb	3n3g	3hkn	2epn	2qi4	1mtr	1g35	1hpx	1ghw
4zx0	1g36	6eif	1xgi	3hkq	4lm3	1dud	4rfd	3gi5	4xip	3iae	4gj3	6d9x
2std	6dil	2w8y	1o33	3igp	1tom	3r88	2p4y	1bai	1k4h	5d48	6b59	2gv7
1a94	2x8z	1d4l	1izh	3n76	2x97	2ya7	6cbg	5upj	4x6o	2xyd	1siv	4q87
3pn1	2qg2	2hnc	4b7r	2hxm	1fjs	6g2l	3rbu	5cjf	1c86	2wc3	3g1d	2qnn
2cn0	5ewa	6b4u	5m7s	5dhu	3d52	1d9i	2o4z	3d91	2qnp	5d45	5sz5	2gyi
5sz0	4ngp	3ekr	5xva	1sgu	2r38	1cgl	1ta6	2zy1	2qbq	3gbb	5dpx	2d3z
4yzu	3s43	2qmg	1rql	4aoi	3d7z	5tt3	4kz3	4ca6	3sut	5dwr	4ks1	4ygf
6g3v	1o2j	1nvs	6c7w	2jjb	4bcm	1cps	2rke	4jsa	3hku	4da5	1f73	4e6d
4lm2	1hyo	2qhy	5eei	5nxg	5jsq	4fsl	3cm2	1c1v	3a9i	4hge	2pvh	5vsj
2xyt	3gta	4zbi	4f09	2q8m	2wlz	4djr	2fqo	4u6c	2xc0	1afl	5nxp	5nxw
3s45	1sqo	5d2r	1gvw	4ngn	3si3	1qbu	2x7u	1v2n	4acc	3d51	3rr4	2q1q
3qwc	5tfx	4llx	2exm	3sm2	1w11	2vvu	4hy1	1hlk	2qbs	1mes	2vnt	4b6p
4qy3	1eoc	4er2	1tmn	2z1w	3hl7	3m3z	4zip	2rkm	3sug	5jfp	1dmp	5l30
4owv	6c0s	1xjd	1f4e	4elf	1pbq	4crf	5sz3	2cf8	2vwm	3vhk	3ckz	1h2k
4ca7	2fqt	3ewj	2i4d	4cwf	5l7h	1uz4	2tmn	5mkr	3aho	5ult	5wlo	1sln
3dx4	5dxt	4cwo	3d4y	2x96	3oim	3l3m	3zbx	1nhz	1ec3	3qfz	4dhl	5dey
2x2r	1ctt	1m2x	5uov	2jh6	1f0t	5l7e	2oxn	4ehz	5v0n	4nuc	5ueu	1x38
3lpk	3s9e	4fs4	1hsh	6h36	4eoh	2y82	4uja	1r1j	6b4l	3djx	1iiq	4gzp
3p17	2i19	1hos	1ppi	2ptz	3gvb	1zoh	3ppm	4e0x	3pbb	1ew8	2xef	2pq9
1grp	5voj	4twp	4uma	3l4z	4p6x	5cep	2i0a	1o5a	3muz	5hi7	4b9k	4afg
5za8	1r1h	1hvr	2ya6	1o5e	4s1g	2a5s	1ec0	3o99	4lhm	2xyf	1v2k	2xda
1f3e	4b9z	1b57	2za5	2rkf	3ibl	2wed	2jfz	3g1v	3bgs	5ime	3pcg	6dj1
3l4u	2vwl	1koj	2gsu	1z95	4jyt	3gc5	2pbw	6eiz	4gr3	2nt7	4mhy	3vtr
1bwa	4fp1	5ula	1sdu	5jxq	1v2j	3qt6	1v48	3u93	4re4	2fu8	2vk6	3u6i
2xnb	3oaf	3o5x	1hp5	3b3x	1pkx	2aoc	4z0q	6c7x	3n4b	6ex1	3hb4	3fx6
6edr	3qgw	4lxd	2pvk	1j36	4bak	2pu1	4ytc	3pb9	4loy	3n3j	1zp8	3bqc
4bc5	1pa9	3d6p	1gjc	2qtt	5d47	3d8z	2wyj	1tng	6f90	4bf6	3ts4	4jzi
3h30	2wzm	1nm6	4j45	2xg9	1gai	1v7a	2wvz	4gj2	4y8x	5uln	4zwx	2usn
2ayr	4ih3	4in9	5wqc	2zxd	3ejp	1a28	6ep4	4e9u	5zag	6std	1ypj	2xhm
4ag8	3vbd	1o2o	2bza	4x24	6f92	1o0h	3ffg	2c3l	1jn4	4qgi	4ual	4m2w
1y3x	3p3g	4xaq	3fas	1x8t	3gqz	3u8n	1h46	1zvx	3t5u	5efh	4efs	1pdz

4cr5	3m37	3k4q	1g98	5c2a	4jia	4wko	4gih	3qqs	5kr2	4u8w	1h23	2w26
1ur9	4kyk	1rnm	5kqy	1m0o	1b8y	4tln	1yq7	5uv2	4zbf	5evb	1qbq	4k18
1d4k	4u69	3nuo	3ckp	4w9o	3tt4	3rv4	5m25	1z71	1b8o	2vh6	4o2b	2o4r
2oc2	5n0e	1c4u	1x1z	1fhd	1wn6	4r76	1t4v	4kxb	2v54	1m2p	3qx5	1lan
1mue	2j4g	3ocz	5oh4	5iwg	2avo	1bq4	2wly	1nq7	1atl	2web	2afx	4ciw
5dh4	4ufk	4mss	2ole	1kug	4wkp	1t31	2jxr	1bhx	1uml	1e5j	4zx4	5eij
3ff3	1lpz	5y12	5sz6	2qi0	3i6o	4llk	5uez	1c5p	3iph	4xiq	5nya	2e94
2qrl	4baq	6b4n	3nu9	4kwg	4mdn	4ap7	2xj7	3djp	4cpy	3eqr	1wvj	1qb1
2qu6	1i2s	3n2p	1n51	3t2q	3bfu	4bcn	4ew3	1cbx	2ra0	1y20	2b7d	1c5t
1o0n	5nw7	2gvj	1a4w	6h34	456c	2d1n	3h89	3aaq	2y80	4elh	5zc5	4zw6
3l0v	4xas	2r3w	4kwo	2v2q	3c2u	4r5b	1wcq	4gfm	5owl	3pe2	3b3s	4msn
1uj5	3djk	4o04	2wm0	2zcq	5g57	5ceq	2r3t	2x7t	2bz6	4eb8	3lpp	2oag
3d4z	3gt9	4dju	1kuk	1wur	1nfx	3g32	3fv1	5nn5	3lvw	1w0z	3ozt	3l4v
5c2h	3zdg	4ryd	1nc1	1ew9	3m36	2pwd	4i72	1lyx	5byi	1bzj	2h15	5k1d
2hhn	5mby	4k0y	7std	6faf	3kgq	1v0k	1u33	3qfy	3l4w	2ojj	1x39	3hp9
1e66	1azm	1kui	2yfx	5t19	2e92	1hi5	4m0e	3sur	2vw1	2oxd	4u6w	1yfz
1ndv	1o5r	1w3l	5cau	1mq5	1gpn	4lm0	3ps1	2cej	2psu	4q7w	4o05	3djv
3nsn	4ax9	2zxg	5oh3	2jew	1ezq	3liw	3wjw	2vkm	4dld	1afk	1sqa	1mmq
1w5v	2p16	1q91	1bv7	3eko	3mhw	3own	3hvj	3ipq	6h5x	5b25	1a30	2xog
1loq	2i4x	3k8c	5sz7	4ykk	2ax9	2w66	6cdl	1zs0	5g1z	2wky	3g5k	2cer
2ojg	1ssq	3nyd	4z83	2bvr	4dv8	1ec9	4ngm	3aau	1yvm	4iid	3hit	5j7w
1ql9	1v0l	3cyw	1d4y	1f8c	1efy	4h42	5mks	4gki	3b4f	2hs1	3ip8	3gdt
2x95	2qwb	5sz4	1trd	4tmn	2am4	5tmn	5l8a	2vsl	1bxr	2f8g	5hz8	3wz8
3g30	6exs	2i4w	4yml	5nxi	2zq0	4m0f	1kzk	2j94	1vso	1qan	1ecq	4cd4
1nwl	3h8b	4ou3	5vb7	1fiv	6cn5	1pr5	6c7q	1ndw	1i37	2zcs	3dx1	2qwf
3acx	3p58	3ewc	2glp	1tpw	5kby	1mfi	6cdj	2vo5	5uoo	2x91	1ppm	4zx1
3utu	4lvt	2z94	2gkl	4gqr	2hoc	4qrh	1hee	3b65	3vdb	1lyb	4wkb	3oe4
4q99	2xpk	1izi	2v77	2e91	1add	8a3h	1qji	1c5x	2a14	3hig	3o5n	3zq9
5mlj	2flr	1sh9	2ceq	4pee	4hdb	2uy0	6dif	2br1	4kiu	5drr	3suu	4ufl
4msc	3pce	4zw5	1c5n	2y5h	3u81	3egt	1ghy	4ish	2ovy	1nc3	2ihj	4ufm
2jh0	4e5w	1g32	3std	6ej3	5c1m	4g90	3rt8	3b3w	3sha	3g35	2xb7	

3b. Test Set for K_i dataset:

2x0y	1uwt	3sww	5upf	6ej2	1gnm	1g2l	1g7g	5vp9	5hct	5ufs	4c2v	4i5c
4cmo	3vd9	1h1s	2bok	3su4	2wgj	2fx6	1pxp	2uwo	4ivb	3mhc	4ei4	3fqe
4mc9	1n5r	4rak	4o97	4r5a	1yp9	3oku	2y8c	1f5k	4u73	3e93	3vfa	3ag9
2bvd	1f4g	3eft	3n7a	5aa9	1jsv	1jq8	3hs4	4cd5	1o5c	1b6l	5sz1	1hvl
5e28	2p95	1k22	3djq	4riv	1d4i	4j46	3bv9	4qll	4sga	2pov	1n4h	3sio
3cl0	1w5w	2vj8	4z1j	4gr8	5c1w	2qhz	2fvd	4f9u	1wc1	3t09	3gv9	2qi7
3zpu	3qgy	3hkt	1i5r	4ufi	3ejq	1ec2	5fcz	1o38	1v2o	4bam	4djx	5n1r
1x8r	1ajv	5a6k	2i4u	4clj	4h3g	3vfb	1g3d	2h4g	1enu	4oc0	4hla	4x6n
3k37	2zda	5sym	3gi6	2bpy	3b5r	4bf1	3iog	2y5g	4gr0	1qbr	3jvr	3gr2
3t60	1owe	1xhy	4fk6	1my4	5mwp	4qf9	4kx8	5tp0	1yqj	2qi6	4mrz	1vfn
3l4y	2oxx	2xbx	3o9e	1q54	2xb8	1qbo	5egm	1ecv	3ldq	1hdq	2zgx	5exn
1oss	4bs0	1z9y	1tcx	4bny	2ihq	5k1f	4bqs	1m2q	5cj6	1jao	4z1k	4kzu
1nvq	4nh7	10gs	4heg	1ppc	2nta	3fvk	2dw7	3suw	1mrn	3ikg	3ehy	5ny3
1e1x	5z5f	4bt4	1gyy	1gyx	2y81	4djv	4q1y	1sdt	4f2w	4cra	4n8q	5zae
4i8w	4crc	2v3d	1usn	1w4o	3bkl	3zc5	1xk9	5kr1	1ndy	5upe	3f7g	4yha
1syi	2avm	2ewb	4dq2	4nue	5l8y	2e2p	5e2k	4few	2r5p	3uw4	1f8e	1h5v
1c83	4a6c	6g3q	1qf0	2iuz	966c	3b68	5e2o	4dsy	3p8o	2j27	4m8x	1tnh
1u0g	3dln	1o2n	6dh1	5cas	5fdc	5kz0	1t5f	3gbe	3e5a	3bl0	1ypg	1ony
5j8z	3q1x	5kr0	5cap	4gii	3t70	6dar	1xug	3zcl	2oxy	3l3l	1o3i	1gnn
2cen	4e3g	1wht	3zxz	3nb5	4crb	4de0	4riu	6evr	3hek	1g3e	3b7r	3str
4bt3	2pvl	3mxd	3lir	1w4p	5exl	4bco	1ogg	3kmc	4n9a	3n0n	2bpv	3zdh
5zaj	1z9g	3su2	1k4g	1pxn	3zj6	5nih	3nu3	2y5f	1tx7	2zmm	2xys	1d4j
2y7x	2i3i	4ban	4j48	1v2t	2rk8	2jke	4ruy	2h6t	1hvh	3s2v	3ozs	2rka
3nu6	2e1w	1f8b	4gzt	3lmk	5dfp	3hll	2d3u	1fpc	3cyx	3ffp	1q84	1qb6
4m8y	2xc4	1y6r	4isi	2uy4	4qd6	5ti0	3f7i					
